# Long-chain fatty acid homeostasis contributes to survival of uropathogenic *E. coli* during copper toxicity

**DOI:** 10.1128/jb.00117-26

**Published:** 2026-03-30

**Authors:** Michael Teve, Braden S. Hanson, Ruhee Marfathia, George Donati, Sargurunathan Subashchandrabose

**Affiliations:** 1 Department of Veterinary Pathobiology, School of Veterinary Medicine and Biomedical Sciences, Texas A&M University 14736, College Station, Texas, USA; 2 Program in Genetics, College of Agriculture and Life Sciences, Texas A&M University 14736, College Station, Texas, USA; 3 Laboratory of Inorganic and Nuclear Chemistry, Wadsworth Center 116287, Albany, New York, USA; University of Virginia School of Medicine, Charlottesville, Virginia, USA

**Keywords:** *E. coli*, UPEC, FabR, FadR, copper, virulence

## Abstract

Uropathogenic *Escherichia coli* (UPEC) is the leading causative agent of urinary tract infection (UTI). Copper (Cu) is a host effector mobilized to the bladder during the innate immune response activated by acute UTI. *E. coli* precisely regulates Cu homeostasis via efflux systems because excess Cu is toxic. Long-chain fatty acid (LCFA) homeostasis is critical for the survival of *E. coli* during envelope stress. However, the interaction between LCFA metabolism and survival during Cu stress, which is also known to induce envelope stress, is not known and is the subject of this report. We hypothesized that fatty acid homeostasis is critical for the survival of UPEC during Cu toxicity and modulates UPEC virulence during UTI. A reverse genetic screen of the KEIO collection identified FabR and FadR as critical proteins for optimal survival of *E. coli* during Cu stress. FadR and FabR regulate transcription genes involved in LCFA metabolism. By using targeted deletion mutants and genetic complementation, we demonstrate that LCFA metabolism affects survival of UPEC during Cu stress. Cell-associated Cu concentrations were decreased in the *ΔfadR* mutant, and we found that the expression of Cu detoxification genes was upregulated in these mutants. UPEC lacking FadR were more sensitive to superoxide stress compared to the wild-type strain. Liquid chromatography-mass spectrometry (LC-MS) analysis revealed that lipid saturation levels were increased in UPEC during Cu stress in a *fabR-* and *fadR*-dependent manner. Furthermore, both mutants displayed lower flagellar expression and attenuated fitness during UTI in the mouse model. Together, our findings demonstrate that LCFA homeostasis is a crucial mediator of Cu homeostasis and UPEC virulence during UTI.

**IMPORTANCE:**

Uropathogenic *Escherichia coli* (UPEC) is the primary causative agent of urinary tract infection (UTI). Previous research has established that UPEC experiences copper stress during UTI, and resistance to copper is critical for UPEC virulence. Envelope stress response systems are activated by copper (Cu) stress, and long-chain fatty acid (LCFA) homeostasis is critical for the survival of *E. coli* during envelope stress. However, the interaction between LCFA metabolism and Cu stress is not known. Our results demonstrate that LCFA homeostasis is a crucial mediator of copper homeostasis in UPEC and modulates UPEC virulence during UTI.

## INTRODUCTION

Uropathogenic *Escherichia coli* (UPEC) is a gram-negative bacterium and the leading causative agent of urinary tract infections (UTIs) (1–3). Uropathogens are the second leading cause of antimicrobial usage leading to the inadvertent rise of antimicrobial resistance, which makes UPEC a prime organism for identifying alternative treatment options for UTI (1–3). The classical paradigm of nutritional immunity has focused on the sequestration of key trace metals like iron, manganese, and zinc (4). Recent studies show that the host also mobilizes trace metals such as copper (Cu) and zinc to induce metal toxicity and restrict pathogen growth as a form of nutritional immunity (5, 6). Multiple groups have shown that Cu is a host effector molecule mobilized to the urinary bladder during UTI as a form of nutritional immunity, a branch of innate immunity (7–11). High concentration of Cu induces toxicity leading to bacterial death through pathways dependent and independent of reactive oxygen species (ROS) (12). UPEC utilizes siderophores to acquire iron and mitigate Cu toxicity where Cu makes initial contact with UPEC at the bacterial membrane interface (5, 6, 8, 9). Cu induces toxicity by disrupting protective membrane layers and interfering with essential cellular processes (13–15). Once inside the cell, Cu homeostasis genes are activated to partition Cu away from the cytoplasm and periplasmic space toward the extracellular space (7, 8, 16). Thus, the cell envelope is a first line of defense against Cu toxicity. The UPEC cell envelope is crucial for survival in host niches (17–21). Bacteria change their membrane compositions in response to stress by inducing changes in the diversity of hydrophilic headgroups and long-chain fatty acid (LCFA) tails (17, 21, 22). Fatty acids are directly incorporated into the phospholipids that form the cell envelope (23–25). Tight regulation of these membrane-integrated fatty acids is necessary for optimal bacterial growth (20–22). However, the interaction between bacterial fatty acid homeostasis and Cu stress is relatively unknown.

Recent reports indicate that fatty acid metabolism contributes to enterohemorrhagic *E. coli* virulence, where supplementation with exogenous fatty acids shows modulation of virulence responses *in vitro* and the presence of intestinal fatty acids leads to induction of the type three secretion system (26–30). Many of these studies were focused on enterohemorrhagic *E. coli* (EHEC) or commensal lab strains of *E. coli* (27, 29). UPEC is endogenous to the gut microflora as a commensal organism where it is exposed to high levels of fatty acids (1, 31). However, the role of fatty acids in UPEC pathogenesis and fitness has not been assessed due to difficulties in measuring fatty acid and lipid content in urine (32).

Here, we utilized the KEIO library to screen non-essential fatty acid metabolism mutants to identify genes contributing to Cu sensitivity and resistance in *E. coli*, followed by investigation in UPEC. We hypothesized that fatty acid metabolism mutants will impair intrinsic resistance to Cu stress in UPEC and attenuate virulence in a murine model of UTI. Our screen revealed that most fatty acid metabolism mutants displayed increased sensitivity to Cu stress. However, a *ΔfadR* mutant UPEC strain exhibited distinct Cu resistance. In this report, we focus on the role of FabR and FadR, transcriptional regulators of fatty acid metabolism, and their contributions to Cu homeostasis and relative fitness of UPEC during UTI.

## MATERIALS AND METHODS

### Media and strains

Lennox lysogeny broth (LB) (tryptone 10 g/L, yeast extract 5 g/L, NaCl 5 g/L, and agar 15 g/L) and M9 minimal media (M9MM) (1× M9 Salts, 2% glycerol, 2 mM MgSO_4_, and 100 mM CaCl_2_) were prepared in Milli-Q water in which Cu was below the level of detection. Reagents used for media preparation were not Chelex-treated, and Cu was added at concentrations indicated in figure legends and results for specific experiments. The flask:medium ratio was maintained at ~4.7 ratio. When required, antibiotics were used with the following concentrations: 100 μg/mL ampicillin, 34 μg/mL chloramphenicol, 50 μg/mL kanamycin, and 10 μg/mL tetracycline. Bacterial cultures were incubated at 37°C, and broth was incubated with shaking at 200 RPM. Chemicals were purchased from Sigma or Fisher Scientific. An initial screen for Cu resistance utilized nine fatty acid metabolism knockout mutants from the KEIO collection (33). UPEC mutants lacking *fabR* and *fadR* were constructed in UPEC strain CFT073 utilizing the Lambda-Red recombineering method (34). UPEC mutants were functionally complemented with plasmids generated from the pGEN-MCS plasmid and endogenous UPEC genes via NEBuilder HIFI Assembly Master Mix (35). Green fluorescent protein (GFP)-promoter fusion plasmids were transformed into UPEC strains via electroporation (36). All strains, plasmids, and primers used in this study are listed in Tables S1 to S3.

### Screen for Cu sensitivity and resistance

Overnight cultures (stationary phase) of wild-type (WT) and fatty acid mutant strains from the KEIO collection were normalized to OD_600_ = 1 and serially diluted 10-fold. Five microliters of each dilution was spotted onto LB agar with 0, 3, or 4 mM CuSO_4_. BW25113 WT strain was added to each plate as a control, and plates were incubated overnight. Strains that exhibited significant resistance or sensitivity compared to the WT were selected for further screening. This assay was repeated with UPEC WT strain CFT073 and isogenic mutants lacking *fabR* or *fadR*. UPEC mutant strains were genetically complemented to confirm activity of FabR and FadR.

### Screen for Cu sensitivity and resistance in M9MM

During the secondary screen, bacterial overnight cultures were diluted 1:1,000 in M9MM containing 0 or 10 μM CuSO_4_. Cultures were then serially diluted 10-fold, inoculated on LB agar. Colony-forming units per mL (CFU/mL) were determined after overnight incubation.

### Trace metal analysis

Inductively coupled plasma-mass spectrometry (ICP-MS) was utilized to determine cell-associated transition metal content (16). Overnight cultures of bacterial strains were diluted 1:100 in fresh LB and incubated for 2 h (mid-exponential phase) at 37°C. Cultures were then split into control cultures and treatment cultures (2 mM CuSO_4_) for 30 min, harvested by centrifugation, and washed thrice with 10 mM HEPES with 0.5 mM EDTA. Cell pellets were weighed for normalization, digested in nitric acid, and diluted to 2% nitric acid with trace element grade water. Levels of Cu, Fe, Zn, and Mn were determined by ICP-MS as we have recently reported (16). This study was conducted with five to six biological replicates, each tested in duplicate.

### Transcriptional reporter assays

GFP-promoter fusion overnight cultures (Table S2) were diluted 1:1,000 in 200 mL of M9MM with kanamycin and incubated for 24 h (stationary phase) at 37°C in black microtiter plates with clear bottoms in media supplemented with or without CuSO_4_. After incubation, OD_600_ and fluorescence (excitation, 485 nm; emission, 530 nm) were determined on an Agilent Cytation5 Plate Reader (36). Relative fluorescence units (RFUs) were blank corrected using empty vector controls and normalized to OD_600_.

### Quantitative PCR

RNA was extracted with RNeasy kits (Qiagen) and used to prepare cDNA libraries with Superscript III reverse transcriptase (Invitrogen). Quantitative PCR (qPCR) was then performed with SYBR Green (Thermo Scientific) in a Bio-Rad CFX Real-Time System with the oligonucleotide primers that we have used earlier (12). Transcripts were normalized to the housekeeping gene *gapA*, and relative expression was determined with the ΔΔCT method using control cultures (no added Cu) of the WT or mutant UPEC strain CFT073 as the baseline (37).

### Assessment of lipid peroxidation

The thiobarbituric acid reactive substance (TBARS) assay was used to assess UPEC lipid peroxidation (38). The assay involves the reaction of lipid peroxidation products, primarily malondialdehyde (MDA), with thiobarbituric acid (TBA), leading to the formation of MDA-TBA. The assay can be measured colorimetrically or fluorometrically, with the fluorometric procedure being more sensitive and used here. Overnight cultures of bacterial strains were diluted 1:100 in fresh LB and incubated for 2 h (mid-exponential phase) at 37°C. Cultures were then split into control cultures and treatment cultures (2 mM CuSO_4_) for 30 min. Cultures were then concentrated into 1 mL of phosphate buffered saline (PBS) and digested per manufacturer instructions (Cayman Chemicals). After digestion, fluorescence (excitation, 485 nm; emission, 530 nm) was determined on an Agilent Cytation5 Plate Reader, and samples were normalized as per manufacturer instructions. To remove potential background interference, the RFU of control samples was subtracted from treated samples.

### Lipid extraction and analysis

Cultures were exposed to Cu as described in trace metal analysis and lipid peroxidation to maintain consistency. For whole cell lipid extraction, cell pellets were resuspended in PBS, and 500 ng/mL of EquiSPLASH (Avanti Polar Lipids) internal standard was added. Lipids were extracted using the method of Bligh-Dyer using a 1:2 ratio of chloroform:methanol (39). The mixture was incubated for 10 min in the dark and then centrifuged at 1,000 × *g* for 10 min at 4°C to separate the organic and aqueous phases. The upper aqueous phase was removed, and the remaining organic phase underwent subsequent lipid extraction. Remaining chloroform was dried, and samples were stored in nitrogen at −80°C until analysis. Lipid composition was analyzed by liquid chromatography coupled to electrospray ionization mass spectrometry (LC-MS) for untargeted lipidomics (40). Mass spectrometry was conducted in normal phase solvents as described previously (41, 42). The chromatograms were evaluated using XCaliber (Thermo Fisher Scientific) and LipidSearch (Thermo Fisher Scientific) to identify lipid species. Study was conducted with three biological replicates in duplicate.

### Lipid data processing

The raw LC-MS data were analyzed on LipidSearch (Thermo Scientific), and raw output was obtained (Table S4). Internal standards were compared to ensure extraction efficiency across samples. Each lipid species was sorted into lipid classes, and relative abundance of each class was calculated by dividing each lipid class by total lipids (Fig. S3). The relative abundance of each lipid molecule was calculated as a ratio of the total lipid content. The lipid molecules were labeled as saturated, monounsaturated, or polyunsaturated, and relative abundance of each saturation state was determined. The relative abundance of each lipid molecule was then evaluated using LIPID MAPS statistical software (43), and volcano plots were generated in GraphPad Prism 10.

### Swimming motility assay

Five milliliters of overnight cultures (stationary phase) of UPEC strains were stab-inoculated into motility agar (1% tryptone, 0.5% NaCl, and 0.25% agar) and incubated at 30°C for 16 h (35). Diameters of zones of swimming motility were recorded. The assays included three biological replicates, with each strain in triplicate.

### Western blot of FliC

Overnight cultures in LB (stationary phase) were adjusted to the same OD and used for immunoblotting. Bacterial cultures were mixed 1:1 with 2× Laemmli Buffer, and equal amounts were loaded into polyacrylamide gels before electrophoresis. One gel was used to transfer to a polyvinylidene difluoride (PVDF) membrane. The second SDS-PAGE gel was stained with Coomassie blue to serve as a loading control. After transfer of proteins, the PVDF membrane was blocked with 2% skim milk, incubated with anti-H1 FliC antibody (1:40,000) prior to incubation with anti-rabbit IgG conjugated to horseradish peroxidase (1:20,000). ECL solution (Cytiva) was applied to the immunoblot for 5–10 min and imaged in a BioRad Chemidoc MP. Images were analyzed in ImageJ v.1.54f 29, and relative quantification was determined using pixel densities.

### Biofilm formation assay

Overnight cultures were diluted 1:100 in 96-well tissue culture-treated polystyrene microtiter plates and incubated at 37°C overnight under static conditions. The microtiter plates were washed with distilled H_2_O, stained with 3% crystal violet, followed by washes to remove excess crystal violet (44). Stained biofilm was dissolved in acetic acid, and OD_590_ was used to quantify biofilm formation.

### Competitive indices

Female CBA/J mice 4–6 weeks of age were used in competitive index experiments as we have reported earlier (8, 12, 45). UPEC strains were cultured overnight in LB, washed and resuspended in PBS to an OD_600_ = 3, and mixed in a 1:1 proportion of WT strain and *fadR* or *fabR* mutant. Mice were anesthetized with Avertin, and 10^8^ CFU of UPEC resuspended in 50 mL of PBS was directly instilled in the urinary bladders using a syringe pump. The syringe pump was operated at a flow rate of 100 mL/min to avoid induction of the vesicoureteral reflux. Urine was collected, and animals were euthanized prior to collection of bladder, kidneys, spleen, and liver after 48 h post-inoculation. Organs were homogenized and bacterial load was determined prior to normalizing CFU counts to milliliters and grams of urine and organs, respectively. Plate counts for WT and mutant strains were enumerated on LB and LB with chloramphenicol. Both the WT and mutant would grow on LB, whereas only the mutants would grow on LB with chloramphenicol. Competitive indices were calculated as a ratio of the CFU counts of mutant to WT in the urine or tissue, and this was normalized to the ratio of the CFU counts of mutant to WT in the inoculum.

## RESULTS

### FabR and FadR are critical for optimal resistance and sensitivity to Cu stress in *E. coli*

**Fig 1:**
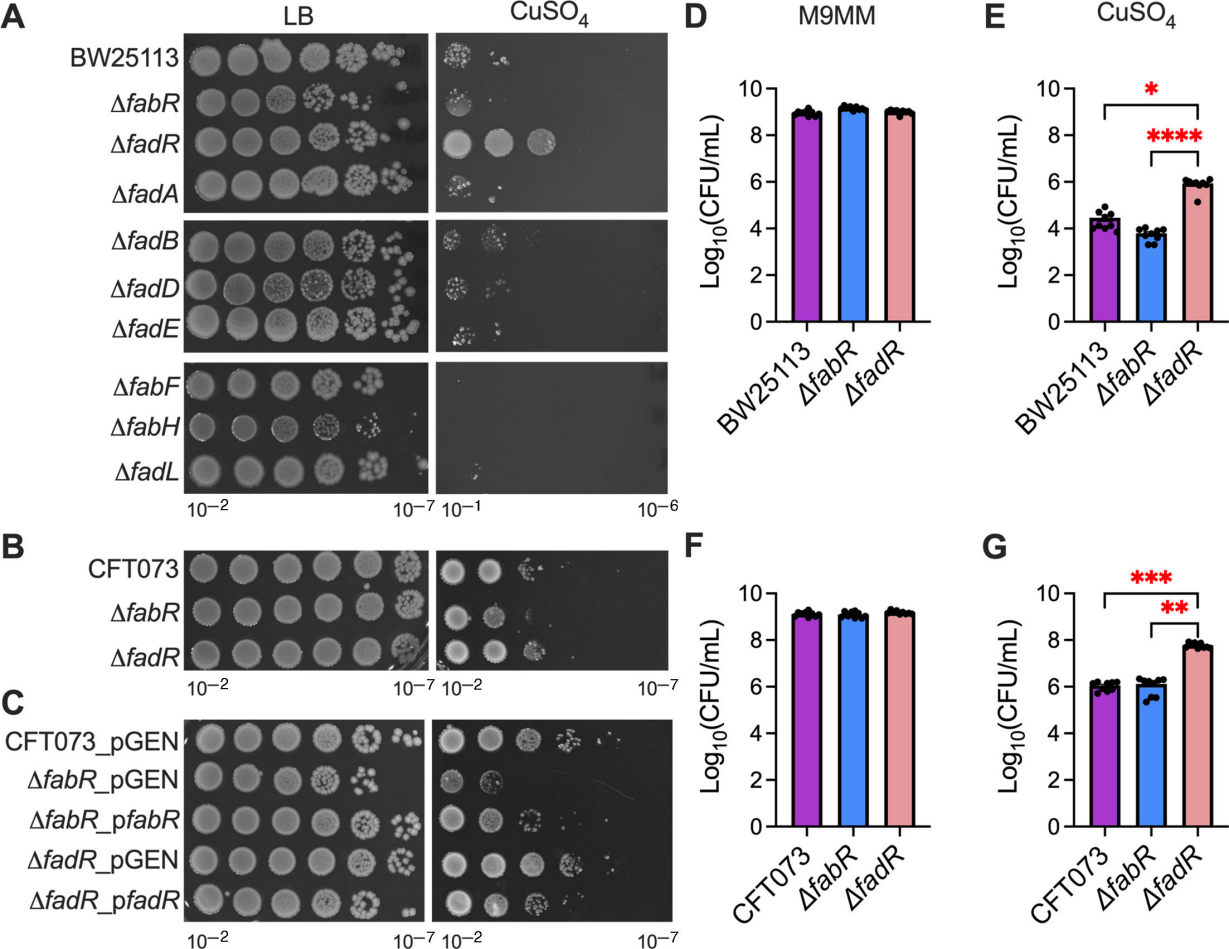
FabR and FadR are critical for optimal survival of during copper (Cu) stress. Serial dilutions of wild-type (WT) and mutant commensal (BW25113) and uropathogenic *Escherichia coli* (UPEC) (CFT073) strains were spot plated on lysogeny broth without and with Cu (**A and B**). Representative images are depicted here (**C**). Representative image of genetically complemented UPEC shows recovery of WT phenotype. Quantification of commensal *E. coli* strains in M9 minimal media (M9MM) without and with 10 μm CuSO_4_ (**D and E**). Quantification of UPEC strains in M9MM without and with 10 μm CuSO_4_ (**F and G**). Assays were conducted in triplicate with at least three technical replicates. Mean and standard error of the mean are plotted here. Kruskal-Wallis test, *P < 0.05, **P < 0.01, ***P < 0.001, and ****P < 0.0001.

Cu is a host effector mobilized to the bladder during acute UTI (7, 9). LCFA metabolism is implicated in the pathogenesis of many bacterial infections (26, 30). However, the impact of Cu stress on LCFA metabolism remains to be determined. LCFA metabolism genes are primarily regulated by fatty acid biosynthesis (*fab*) and fatty acid degradation (*fad*) genes. The KEIO collection has nine mutants in the Fab and Fad pathways. Here, we took a reverse genetic approach by utilizing the KEIO collection to screen the nine fatty acid metabolism mutants in laboratory/commensal *E. coli* strain on Cu-supplemented medium (Fig. 1A). Compared to WT, the *ΔfadR* mutant was the only mutant to exhibit a significant increase in resistance to Cu stress, whereas the *ΔfabR* mutant was significantly more sensitive to Cu stress (Fig. 1A). The *ΔfadB*, *ΔfadD*, and *ΔfadE* mutants exhibited no difference in viability during Cu stress compared to WT, and the *ΔfabF*, *ΔfabH*, and *ΔfadL* mutants were more sensitive to Cu stress compared to WT and the *ΔfabR* mutant (Fig. 1A). FadR and FabR are transcriptional regulators of LCFA biosynthesis and degradation genes in *E. coli* and other bacteria (46–50). FadR inhibits the transcription of LCFA degradation genes and activates the transcription of LCFA biosynthesis genes (51). FabR inhibits transcription of LC fatty acid biosynthesis genes (49). The involvement of FadR and FabR in Cu homeostasis in UPEC was further verified with targeted Δ*fabR* and Δ*fadR* mutations generated by Lambda Red Recombineering and genetically complemented mutants (Table S1). UPEC mutants were screened for Cu sensitivity and displayed comparable phenotypes to the mutants in the commensal *E. coli* background during Cu stress (Fig. 1B). Furthermore, genetic complementation of each mutant reverted the growth during Cu stress to wild-type levels (Fig. 1C).

Since we observed qualitative differences in *E. coli* survival during Cu stress, we next tested the extent to which Cu affects the viability of mutants defective in regulation of LCFA metabolism. We used supraphysiological levels of Cu in the qualitative screens since the assays were performed in a rich medium (LB, Fig. 1A through C) (16). Physiological levels of Cu at infection sites are much lower (25 μM), with select sites such as the gall bladder and gut having higher levels of Cu (~2 mM) than the concentrations used in our primary screen (52). Therefore, we transitioned to M9 minimal media (M9MM) with 2% glycerol as the sole carbon source. This allowed us to decrease the concentration of Cu to 10 μM, which is closer to physiologic levels. WT and mutant *E. coli* were cultured overnight in M9MM with or without 10 μM of Cu prior to determining viable counts. Consistent with our earlier results, commensal *ΔfadR* mutant displayed significant increase in survival compared to the WT strain and *ΔfabR* mutants in M9MM containing 10 μM Cu (Fig. 1E). Importantly, the UPEC *ΔfadR* mutant displayed increased resistance to Cu compared to WT and *ΔfabR* mutant (Fig. 1G). Overall, commensal and UPEC strains behave similarly during Cu stress, with UPEC strains displaying inherent Cu resistance compared to commensal strains. In summary, our results show that FabR and FadR contribute to Cu resistance and sensitivity in *E. coli,* respectively.

### Cell-associated Cu content is decreased in mutants with defects in LCFA homeostasis

**Fig 2:**
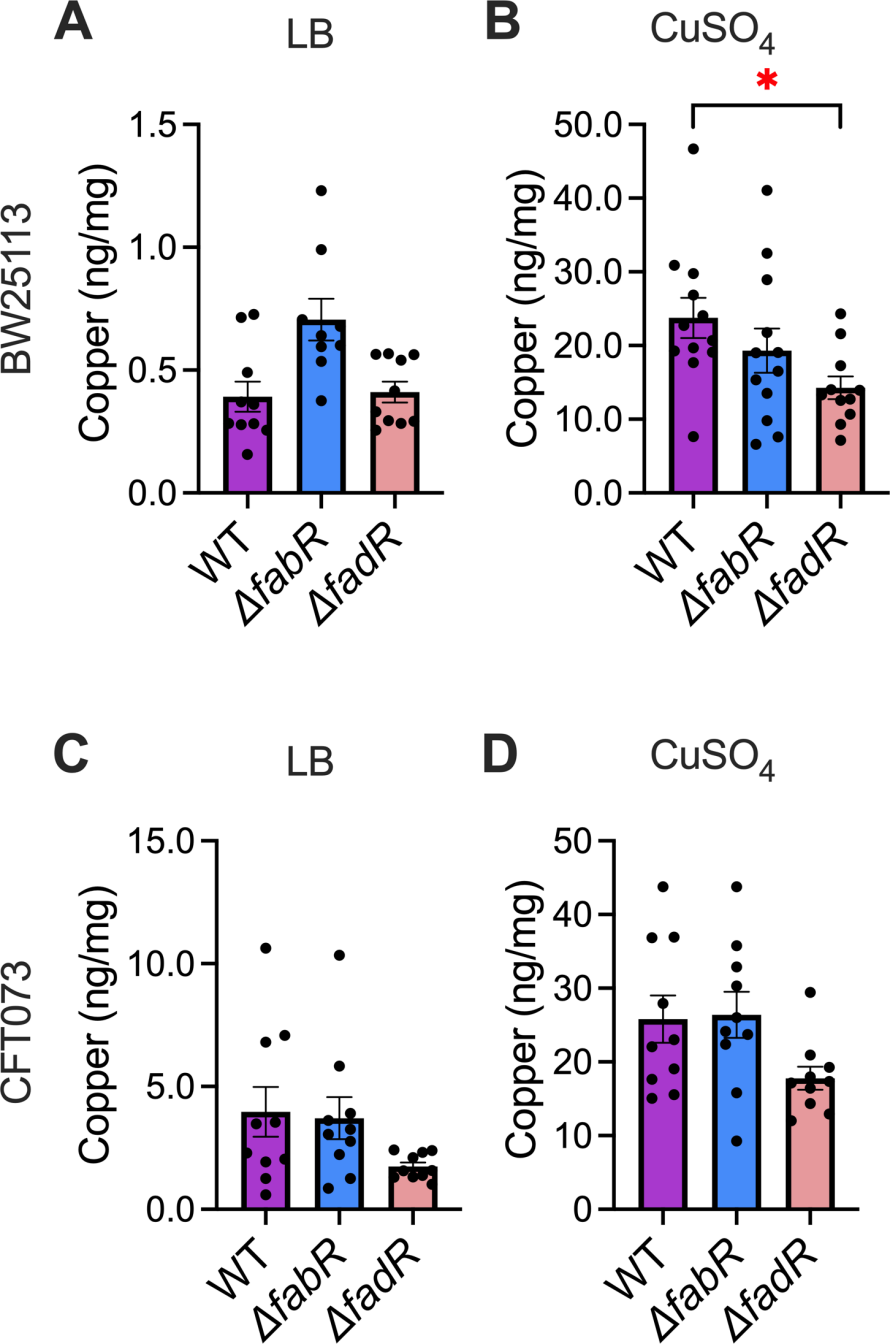
Cell-associated copper (Cu) content is decreased in Δ*fadR* mutants. Wild-type (WT) and mutant commensal strains were cultured in lysogeny broth (LB) without and with Cu (**A and B**). WT and mutant uropathogenic *Escherichia coli* (UPEC) strains were cultured in LB without and with Cu (**C and D**). Cell-associated Cu content was determined by inductively coupled plasma-mass spectrometry. Mean and standard error of the mean are plotted. (*N* = 6 for commensal and *N* = 5 for UPEC, in duplicate.) Analysis of variance, *P < 0.05.

To test whether mutant strains are compromised in their ability to maintain normal levels of Cu, trace metal content was determined by inductively coupled plasma-mass spectrometry (ICP-MS). Commensal and UPEC strains were cultured in LB media and stressed with 2 mM Cu, a subinhibitory concentration of Cu (16). We used LB in this assay since the mutants display a Cu-responsive phenotype in LB (Fig. 1A and B), to collect cell pellets in mg range to normalize ICP-MS data, and because of our interest in relative fitness of these mutants compared to the parental WT strain. Cu levels were increased in WT and mutant strains incubated in Cu-supplemented media (Fig. 2B and D). There was no statistically significant difference in the Cu content of WT, *ΔfabR*, or *ΔfadR* strains grown in LB alone, possibly due to the range of concentrations detected in this assay (Fig. 2A and C). The commensal *ΔfadR* mutant displayed a statistically significant decrease in cell-associated Cu relative to commensal WT and *ΔfabR* mutants treated with Cu (Fig. 2B). Our UPEC *ΔfadR* mutant trended toward decreased Cu concentration (Fig. 2C). We expected *ΔfabR* mutants to have increased Cu content due to their increased sensitivity to Cu stress. Surprisingly, we observed Cu levels similar to WT for commensal and UPEC strains (Fig. 2B and D). We have previously reported that Cu stress leads to dysregulation of Fe, Mn, and Zn homeostasis in commensal and pathogenic *E. coli* strains (12, 16). Levels of these important transition metals were also determined during Cu stress (Fig. S1A through F) for the LCFA mutants. The UPEC *ΔfadR* mutant had decreased accumulation of zinc both in the presence and absence of Cu stress (Fig. S1F).

### FabR and FadR contribute to transcriptional activation of Cu homeostasis systems

**Fig 3:**
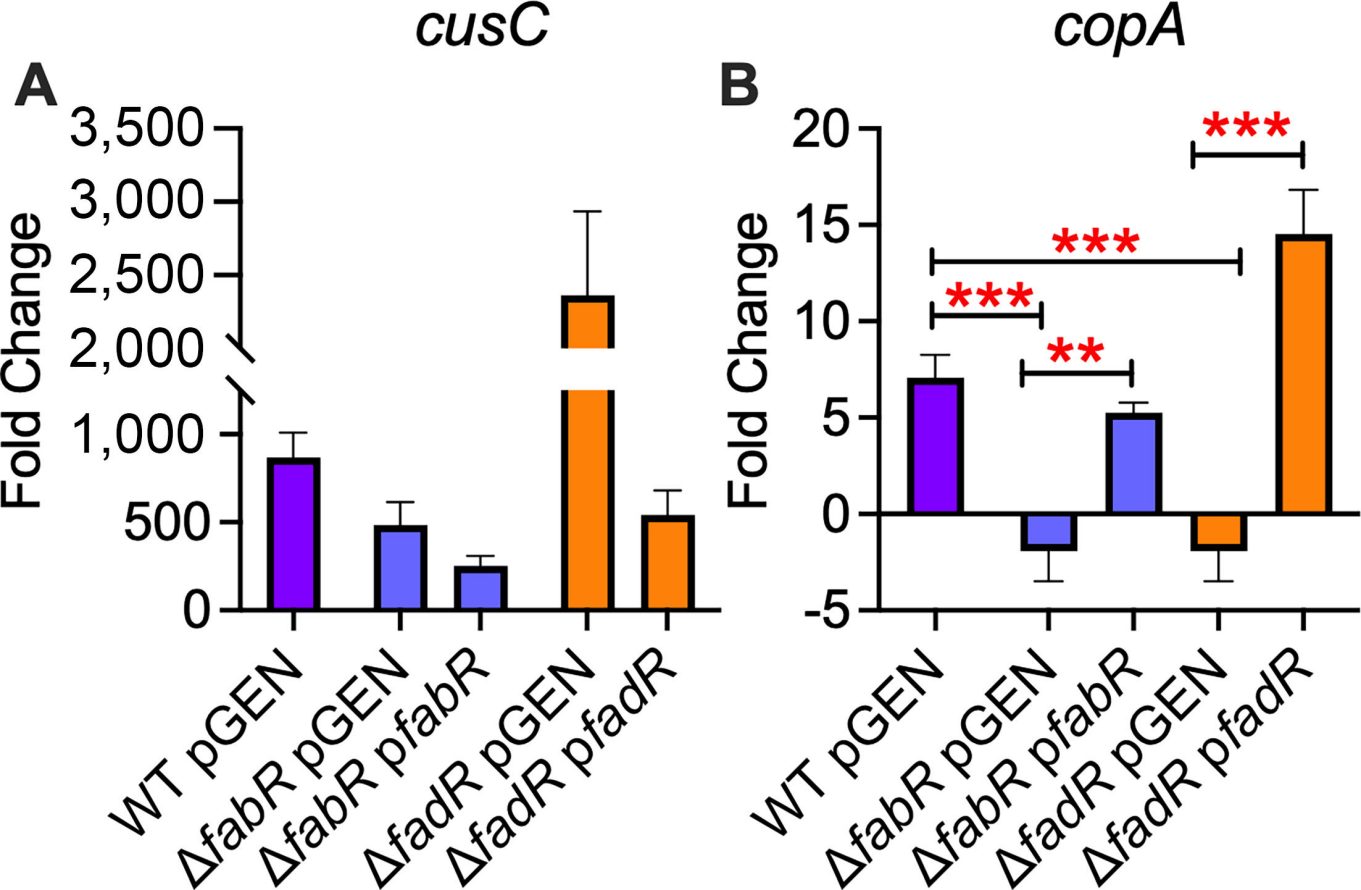
Regulators of fatty acid metabolism affect CueR-regulated *copA* transcription in uropathogenic *Escherichia coli* (UPEC). Wild-type (WT), mutant, and complemented UPEC strains were cultured in M9 minimal media with or without 5 μM copper (Cu). Fold change in expression of CusR-regulated *cusC* (**A**) and CueR-regulated *copA* (**B**) relative to control (no Cu) cultures was determined by quantitative PCR. Mean and standard error of the mean are plotted. Analysis of variance, **P < 0.01 and ***P < 0.001.

*E. coli* has highly conserved Cu homeostasis systems regulated by CusR and CueR, Cu-responsive transcription factors (7). CusR is part of the two-component Cu regulatory system, and CueO contributes to periplasmic metal tolerance (7, 53–56). During UTI, expression of CusR and CueR regulated genes is significantly upregulated because of the mobilization of Cu to urine (8). To determine the effects of FabR and FadR on Cu homeostasis, we performed qPCR and transcriptional reporter assays with plasmids containing *cusR* and *cueO* promoters fused to the green fluorescent protein (GFP) gene that were transformed into WT and mutant UPEC strains (Tables S1 and S2). Strains were incubated overnight in M9MM with or without Cu prior to determining transcript abundance by qPCR or GFP fluorescence. Cu treatment resulted in significant upregulation of CusR-regulated *cusC* transcript levels in wild-type and mutants, compared to control cultures (Fig. 3A). However, there were no genotype-specific differences in expression (Fig. 3A). Cu treatment resulted in significant upregulation of CueR-regulated *copA* transcript levels in wild-type and complemented mutants, compared to control cultures. However, there was a significant decrease in *copA* abundance in the mutants, and this defect was rectified in the complemented mutants (Fig. 3B). GFP reporter assays revealed that treatment with Cu resulted in significantly increased CusR-regulated *cusR* and CueR-regulated *cueO* transcription in *ΔfabR* mutants compared to the untreated controls (Fig. S2). Treatment with Cu resulted in significantly increased *cusR* but not *cueO* transcription in *ΔfadR* mutants compared to the untreated controls (Fig. S2). Our results indicate a dichotomy in interaction between transcriptional regulators of LCFA metabolism and Cu efflux/detoxification.

### Loss of *fadR* protects against superoxide stress and lipid peroxidation

**Fig 4:**
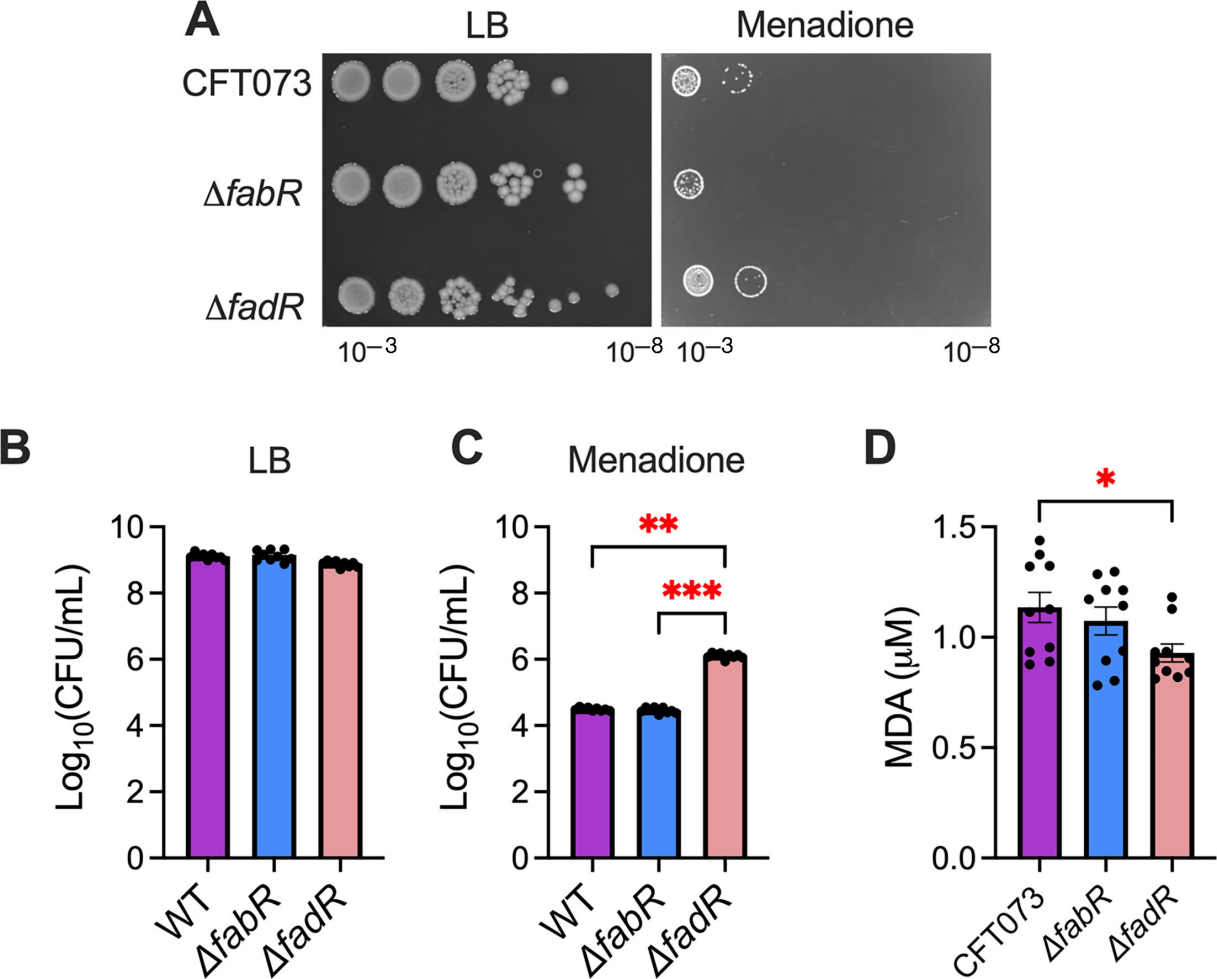
Loss of FadR promotes resistance to superoxide stress. (**A**) Serial dilutions of wild-type (WT) and mutant uropathogenic *Escherichia coli* (UPEC) (CFT073) strains were spot plated on lysogeny broth (LB) with or without 5 mM menadione. A representative image is depicted here (**A**). WT and mutant UPEC strains were cultured overnight in LB broth (**B**) and LB with 7.5 mM menadione (**C**). CFU were enumerated and compared between strains. Assays were conducted in triplicate with at least three technical replicates. Kruskal-Wallis test. **P < 0.01 and ***P < 0.001. Lipid peroxidation was measured via thiobarbituric acid reactive substance assay from UPEC cultures in exponential phase from LB supplemented with 2 mM CuSO_4_ (**D**). Lipid peroxidation of UPEC WT and mutants during 2 mM copper (Cu) stress. Data were normalized to LB cultures without Cu supplementation. Mean and standard error of the mean are plotted. Analysis of variance, *P < 0.01.

Cu is a known catalyst for reactive oxygen species and superoxide stress (12, 53–56). We screened our WT and mutant UPEC strains against hydrogen peroxide and menadione (superoxide generator) stress. Menadione supplementation reveals that *ΔfadR* mutants have increased resistance to superoxide compared to WT and *ΔfabR* mutant strains (Fig. 4A and C). In contrast, *ΔfabR* mutants showed increased sensitivity to menadione (Fig. 4A). We want to note that there was a concentration-dependent response to superoxide sensitivity in the *ΔfabR* mutant compared to the parental strain (Fig. 4A vs C). These differences could have arisen from growth conditions on agar and broth and the technical limitation that prevented using the same concentration of menadione under both growth conditions. However, *ΔfabR* and *ΔfadR* mutants have no significant change in viability on hydrogen peroxide-containing media compared to WT UPEC (Fig. S3). Furthermore, Cu and free radicals are known inducers of lipid peroxidation (57–60). Therefore, we conducted the thiobarbituric acid reactive substance (TBARS) assay to determine lipid peroxidation levels of *ΔfabR* and *ΔfadR* mutants at baseline and during Cu stress. WT and mutant UPEC strains were cultured to the log phase in LB and exposed to 2 mM Cu for 30 min. The production of malondialdehyde (MDA), a byproduct of lipid peroxidation, was quantified. Decreased levels of lipid peroxidation were observed in the *ΔfadR* mutant, compared to the WT strain (Fig. 4D). Collectively, the *ΔfadR* mutant strain exhibits increased resistance to superoxide and decreased lipid peroxidation in the presence of Cu.

### Changes in relative abundance of lipids during Cu stress

**Fig 5:**
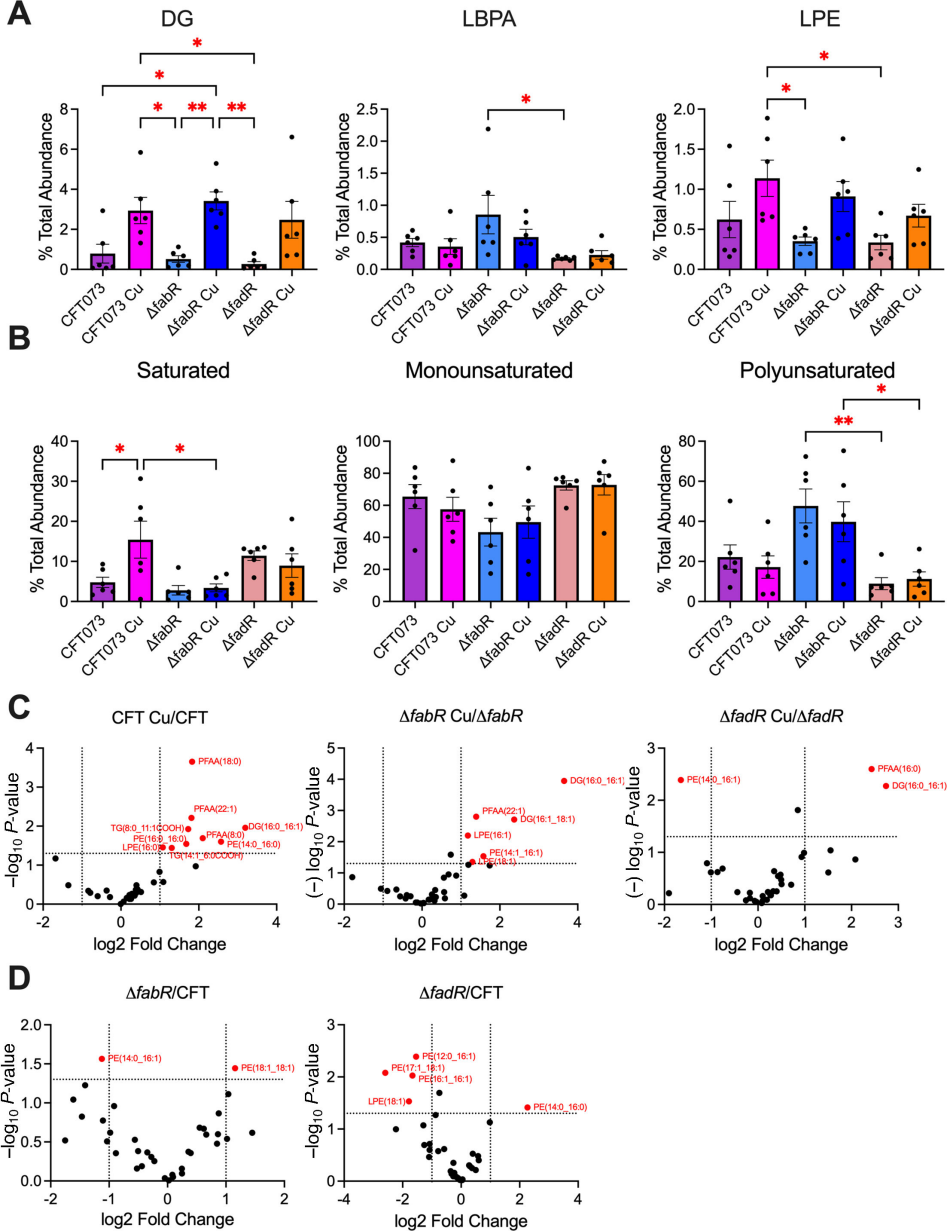
Changes in lipid saturation levels during copper stress. (**A**) Relative abundance of lipid classes. Lipid class areas were normalized to total area. Mean and standard error of the mean (SEM) are plotted. (**B**) Relative abundance of lipid saturation state. Identified lipids were labeled as saturated, monounsaturated, polyunsaturated, or coenzyme, and average relative abundance was determined for each biological sample and summed into their level of saturation. Mean and SEM are plotted. (**C and D**) Volcano plots of relative abundance of lipid species. Statistical analysis was performed on LIPID MAPS and graphed in GraphPad Prism 10. Lipid classes and saturation states underwent analysis of variance analysis with Tukey’s multiple comparisons. *P < 0.05 and **P < 0.01.

To measure changes in lipid species under Cu stress, WT and mutant UPEC strains were cultured in LB and exposed to Cu. Whole cell lipid extracts were analyzed using LC-MS coupled to electrospray ionization (ESI). Positive ESI returned 247 lipid molecules, and negative ESI returned 152 lipid molecules (Table S4). For both modes, phosphatidylethanolamine (PE) was the most abundant lipid class (~90% of all lipids) followed by phosphatidylglycerol in negative ESI mode (Fig. S4). Furthermore, the positive ESI mode also showed statistically significant increase in diacylglycerols (DG) and lysophosphatidylethanolamine (LPE) during Cu stress (Fig. 5A). Lipid species that were absent in ≥80% of samples were removed from further analysis. This retained 35 lipid species and 26 lipid species for positive and negative ESI runs, respectively. Results from positive ESI mode were used for the remainder of this study. During Cu stress, the abundance of saturated lipids increased for WT UPEC but not mutants (Fig. 5B). Furthermore, the *ΔfabR* mutant exhibited decreased saturated lipid abundance compared to WT (Fig. 5B). Meanwhile, the *ΔfadR* mutant exhibited decreased polyunsaturated lipid abundance compared to the *fabR* mutant (Fig. 5B). Relative abundance of individual lipid species was determined in LIPID MAPS (43) and depicted in volcano plots (Fig. 5C and D). During Cu stress, WT UPEC exhibited increased abundance of six saturated and three monounsaturated lipid species (Fig. 5C). Cu-treated *ΔfabR* mutants exhibited increased abundance of four monounsaturated lipid species and two polyunsaturated lipid species compared to untreated controls (Fig. 5C). Cu-treated *ΔfadR* mutants had increased abundance of perfluoroalkyl acid (16:0) and DG (16:0_16:1) and decreased levels of PE (14:0_16:1) (Fig. 5C). The *ΔfabR* mutants exhibited increased levels of PE (18:1_18:1) and decreased levels of PE (14:0_16:1) compared to WT (Fig. 5D). The *ΔfadR* mutants exhibited decreased abundance of saturated lipid species and increased abundance of mono- and polyunsaturated lipid species relative to WT (Fig. 5D). There were no changes in lipid classes in +ve and −ve ESI modes, acyl chain saturation, coenzyme, and monounsaturated fatty acid levels caused by Cu treatment or loss of FabR or FadR (Fig. S4 and S5). Overall, FabR and FadR are involved in regulating the saturation state of lipid acyl chains, and treatment with Cu alters the lipid composition of WT UPEC.

### Role of LCFA metabolism in the expression of flagella and biofilm formation

**Fig 6:**
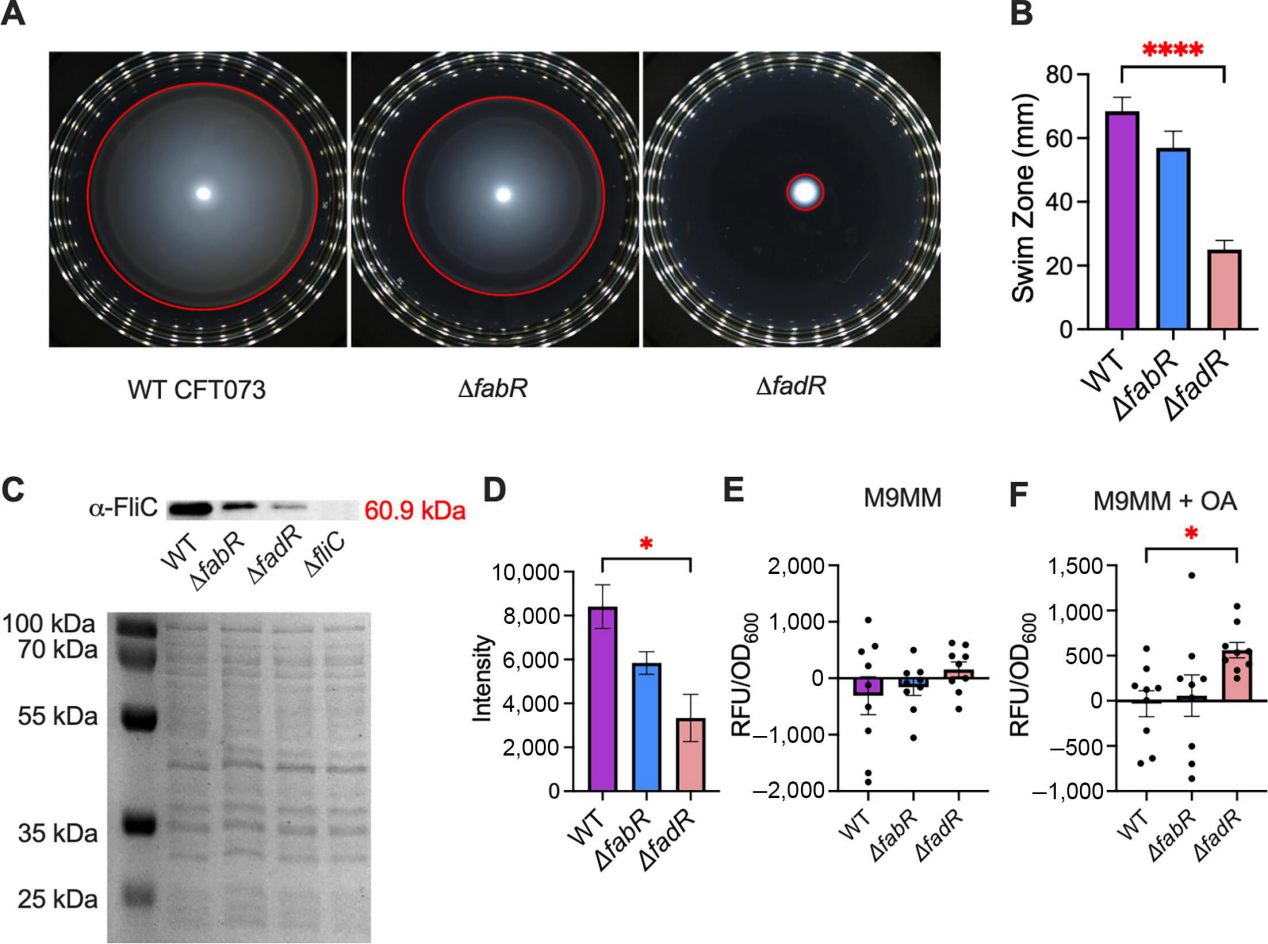
FabR and FadR are involved in regulating swimming motility. Representative image of zones of swimming motility of wild-type (WT) uropathogenic *Escherichia coli* (UPEC), Δ*fabR*, and Δ*fadR* mutant in soft agar (**A**). Red circles indicate the widest area of swimming. Quantification of swim zones of WT UPEC and mutants in soft agar (**B**). Levels of flagellin (FliC) were determined by immunoblotting, and the Δ*fliC* mutant was used as a negative control. A representative blot of FliC is depicted here with SDS-PAGE as loading control (**C**). Images from five independent experiments were used to quantify signal intensity (**D**). Transcriptional activity of the *fliC* promoter was determined using a green fluorescent protein reporter in M9 minimal media (M9MM) alone (**E**) and supplemented with 300 μM oleic acid (**F**). Mean and standard error of the mean are plotted here. *P < 0.05, ****P < 0.0001, analysis of variance with Tukey’s multiple comparisons test.

**Fig 7:**
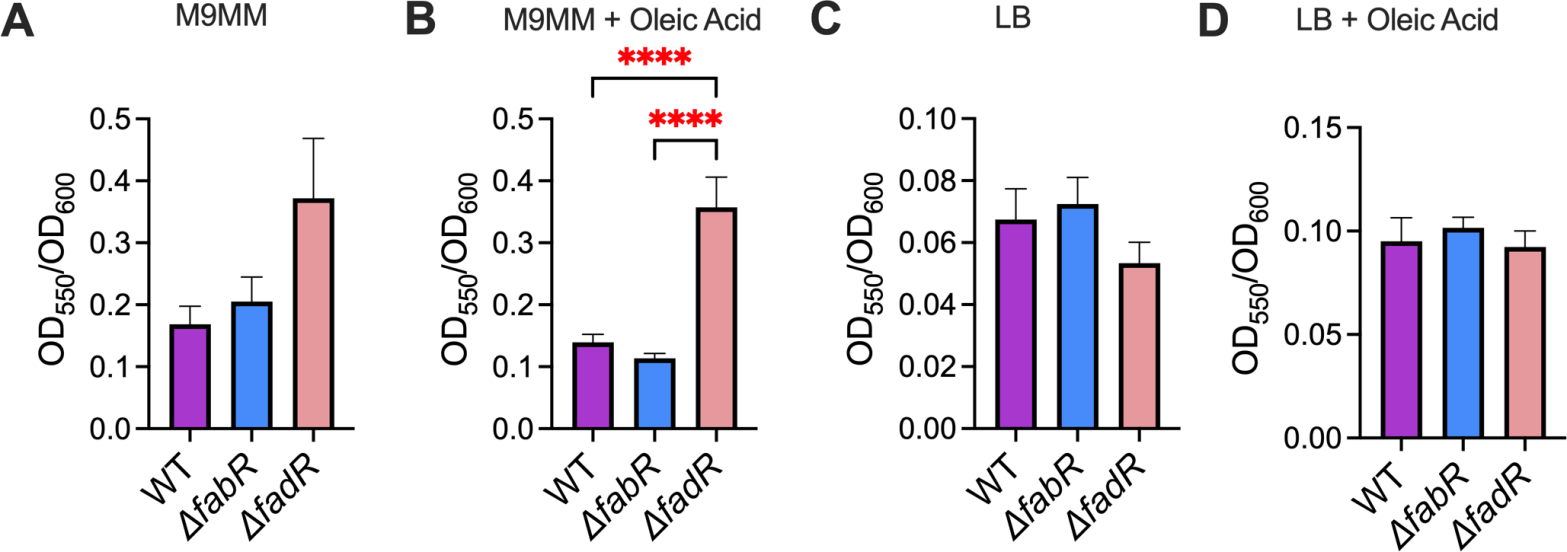
FadR affects biofilm formation in M9 minimal media (M9MM). Biofilm formation was assessed using a crystal violet assay to measure the biomass. Wild-type (WT) and mutant uropathogenic *Escherichia coli* strains were incubated in M9MM without or with oleic acid (**A and B**) or in LB without or with oleic acid (**C and D**) statically for 24 h at 37°C prior to staining with crystal violet. Biomass was normalized to the optical density of cultures. Mean and standard error of the mean are plotted. Analysis of variance, ****P < 0.0001

Given the importance of LCFA metabolism on envelope homeostasis and the known links between envelope homeostasis, flagellar motility, and biofilm formation, we investigated the virulence-associated phenotypes of UPEC described here. Swimming motility is critical for optimal fitness of UPEC during UTI (31, 35, 61, 62). Our swimming motility assays revealed that the *ΔfadR* strain exhibited a significant defect in swimming motility compared to WT and *ΔfabR* mutant UPEC strains (Fig. 6A and B). We then tested whether the decreased motility was due to changes in metabolic status using resazurin as an indicator for metabolic activity. The *ΔfadR* mutant exhibits significantly higher levels of resazurin reduction than WT UPEC and the *ΔfabR* strain, ruling out this possibility (Fig. S6). We then determined the levels of flagellin production by immunoblotting, which revealed a significant decrease in FliC levels in the *ΔfadR* strain (Fig. 6C and D). Promoter activity was measured by utilizing plasmids containing the *fliC* promoter fused to GFP. Strains were incubated overnight in M9MM with or without 300 µM oleic acid prior to fluorescence and OD_600_ determination. The screen revealed that the *ΔfadR* mutant exhibited enhanced transcription when exposed to oleic acid (Fig. 6E and F). Motility assays, immunoblots, and transcriptional reporter assays were performed in motility agar, LB broth, and M9MM, respectively. Collectively, our results paint a picture of no change in *fliC* transcript abundance but decreased FliC protein and poor swimming motility. We then studied the biofilm-forming potential of WT and mutant UPEC strains through a crystal violet biofilm assay (44). Surprisingly, *ΔfadR* mutants displayed higher biofilm-forming potential compared to the WT UPEC strain in M9MM supplemented with 300 mM oleic acid (Fig. 7).

### FabR and FadR contribute to UPEC fitness during UTI

**Fig 8:**
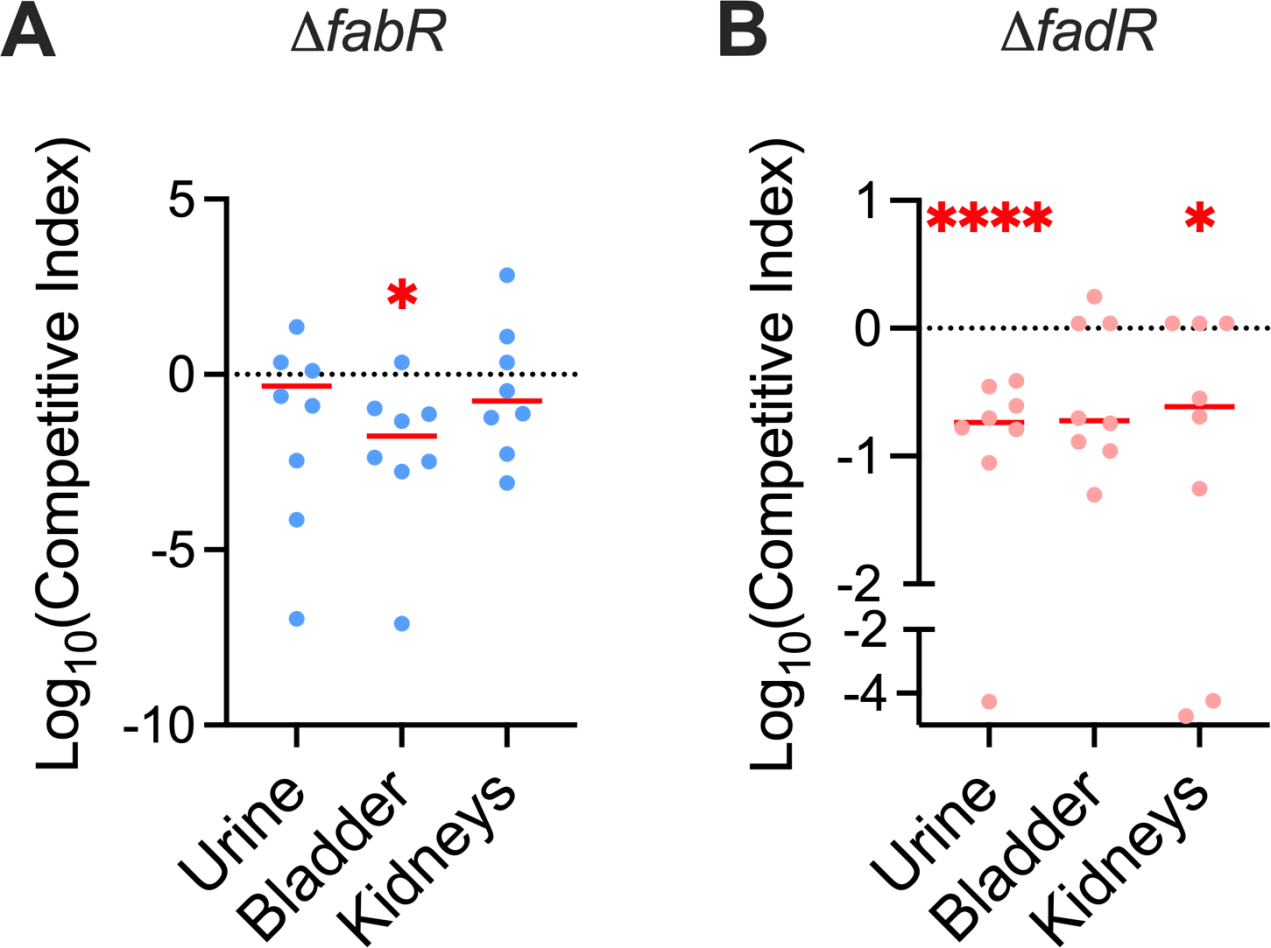
FabR and FadR contribute to uropathogenic *Escherichia coli* (UPEC) fitness in the mouse model of urinary tract infection. Competitive index of *δfabr* (**A**) and *ΔfadR* (**B**) relative to wild type (WT). Mixtures of WT and mutant UPEC strains in a 1:1 ratio were inoculated in the bladders of female CBA/J mice. CFUs were enumerated, and the competitive index was calculated as the ratio of the mutant strain to the WT strain and normalized to the ratio of the inoculum. Each symbol corresponds to results from one mouse, and bars indicate the median. The dotted line indicates no deviation to fitness (competitive index = 1). *N* = 8 for each mutant. Wilcoxon signed-rank test. *P < 0.05 and ****P < 0.0001

Next, we investigated whether FabR and FadR are involved in UPEC fitness in the murine model of UTI. Adult female CBA/J mice were co-inoculated with equal numbers of WT UPEC and *ΔfabR* or *ΔfadR* strain in a 1:1 ratio. We used a co-infection assay to directly compare the fitness of mutants relative to the parental strain and to avoid inherent variation in urinary tract colonization between animals requiring the need for a higher number of animals to be used. Bacterial burden in urine, bladder, and kidneys was assessed at 48 hours post-infection (hpi) and used to calculate competitive indices as described in *Materials and Methods*. The *ΔfabR* mutant exhibited significantly decreased fitness in the bladder relative to the parental strain, and the *ΔfadR* mutant exhibited significantly decreased fitness in urine and kidney samples (Fig. 8). There was no significant difference in the fitness of *ΔfabR* and *ΔfadR* mutant strains in LB, liver or spleen (Fig. S7A through C). Overall, the median fitness values of *ΔfabR* and *ΔfadR* mutants trended toward reduced fitness in the urinary tract compared to WT UPEC.

## DISCUSSION

Prior research from our laboratory and colleagues has shown that Cu is a host effector molecule mobilized to the urinary bladder during UTI as a form of nutritional immunity, a branch of innate immunity (7–11). Cu makes initial contact with UPEC at the bacterial membrane interface where UPEC utilizes siderophores to acquire iron and mitigate Cu toxicity (7, 10, 16, 63). The cell envelope is the first line of defense against Cu and is primarily composed of phospholipids and proteins in a fluid mosaic model (18, 20, 21). A variety of environmental stressors and conditions leads to remodeling of bacterial phospholipids where variation in the phospholipid composition and membrane integrity is determined by the composition of LCFAs incorporated as hydrophobic acyl chains (18, 20, 21, 64). However, not much is known about the role of fatty acids during Cu stress. Here, we utilized the KEIO library to screen non-essential fatty acid metabolism mutants to identify genes contributing to Cu sensitivity and resistance in *E. coli*. We hypothesized that the fatty acid metabolism mutants will impair the intrinsic resistance of UPEC to Cu stress. Our screen revealed that most fatty acid metabolism mutants displayed increased sensitivity to Cu stress. However, the *ΔfadR* mutant exhibited significantly enhanced resistance to Cu. In this report, we focus on the role of FabR and FadR, transcriptional regulators of fatty acid metabolism, and their contributions to Cu homeostasis and UPEC fitness.

FabR is a transcriptional repressor of fatty acid biosynthesis in *E. coli* (49–51, 65). FadR is a global transcriptional activator of fatty acid biosynthesis and repressor of fatty acid degradation in *E. coli* (18, 48, 50, 51). Our results indicate a role for these proteins in Cu homeostasis in both UPEC and commensal *E. coli*. Trace metal analysis showed that there was a decrease in the Cu content of the *ΔfadR* mutant relative to WT. This suggests that loss of FadR compromises mechanisms involved in Cu import and/or promotes efflux and detoxification pathways. To further investigate this decrease in Cu concentration, we utilized GFP-fusion reporters to study transcriptional regulation of known Cu efflux systems in UPEC. Our results show that *cusR* is significantly upregulated in the *ΔfadR* mutant compared to the WT UPEC strain. Therefore, the decreased levels of Cu seen in our trace metal analysis studies can be attributed to increased expression of the Cus Cu efflux system, highlighting the connection between Cu homeostasis and LCFA metabolism. Thus, FadR is involved in modulating the expression of the CusRS regulatory system and the CusCFBA efflux system leading to increased sensitivity to Cu stress. Furthermore, the *ΔfabR* mutant did not display significant deviation in Cu content relative to the WT strains. This can be explained by the upregulation of both CusR and CueO in the *ΔfabR* mutant. CueO is a periplasmic Cu oxidase that converts the more toxic Cu (I) to less toxic Cu (II), where it is then exported from the cell by the CusCFBA system (7, 55, 66). A high level of induction of *cueO* by the *ΔfabR* mutant may explain the lack of deviation from WT since it can lead to increased cell-associated Cu content as opposed to the *ΔfadR* mutant, which has only increased expression of *cusRS* and *cusCFBA* (54–56). Together, these results suggest that FadR and FabR play a role not just in LCFA homeostasis but are also interlinked with maintenance of optimal Cu concentration in *E. coli*.

Urothelial cells and phagocytes produce reactive oxygen species (ROS) to expunge UPEC during infection (67). We assayed the *ΔfabR* and *ΔfadR* mutants for survival under peroxide and superoxide stress to determine effects of ROS stress on these mutants. The screen revealed that *ΔfabR* and *ΔfadR* mutants do not display any growth differences on H_2_O_2_-supplemented media compared to WT. However, *ΔfadR* mutants display a significant resistance to menadione, an intracellular superoxide generator. Thus, FadR contributes to Cu and superoxide sensitivity but not to peroxide stress. Cu is historically thought to be a generator of reactive oxygen species through Fenton and Haber-Weiss reactions (68, 69). However, recent studies have challenged this paradigm in *E. coli* and UPEC (15). The presence of bladder-produced ROS increases malondialdehyde, a byproduct of lipid peroxidation, in urine of acute UTI patients (59, 67). The standard model of lipid peroxidation requires ROS to attack alkene bonds in polyunsaturated fatty acids (PUFAs) to start the oxidative chain reaction (60, 70). However, bacteria such as *E. coli* primarily produce saturated or monounsaturated fatty acids (70, 71). LCFA metabolism regulators FabR and FadR are involved in membrane lipid homeostasis by modulation of their LCFA tails (49, 51, 64, 65, 72). FabR regulates the ratio of unsaturated fatty acids in the membrane, and a *fabR* mutant increases the proportion of unsaturated fatty acids in the membrane (49, 64). Deletion of FadR significantly decreases unsaturated fatty acid levels in *E. coli* (51, 65). Therefore, we measured bacterial lipid peroxidation during Cu stress to determine if lipid peroxidation has a role in *ΔfabR* and *ΔfadR* mutant sensitivity and resistance to Cu toxicity. Here, we demonstrate using the TBARS assay that the *ΔfadR* mutant has significantly decreased lipid peroxidation compared to WT during Cu stress. This may indicate that the *ΔfadR* mutant resistance to Cu toxicity is due to decreased lipid peroxidation. This decrease in lipid peroxidation could be due to the decrease in unsaturated fatty acids levels in the *ΔfadR* mutant. The TBARS assay is considered a standard practice for quantifying lipid peroxidation, but there are limitations to its metabolite specificity and inconsistent yield when analyzing biological systems (73). Therefore, it is recommended to follow up TBARS assays with supplementary assays.

Cu affects membrane permeability and homeostasis of eukaryotic cells (10, 71, 72). In eukaryotic systems, Cu is proposed to be involved in disruption of membrane fluidity through formation of diCu-coupled phospholipid adducts and increases in lipid oxidation by Cu-bound PE (71, 72). Therefore, we conducted LC-MS to identify changes in lipid species of WT and mutant UPEC strains during Cu stress. We would like to note that the comprehensive UPEC lipid composition described here for the first time will be a useful resource for researchers. To determine if we can identify changes in lipid classes, we determined the relative abundance of each lipid class relative to the total lipid content. PE is the most abundant lipid class in the UPEC cell, accounting for ~80% of all identified lipid species. Our studies indicate an overall increase of DG and LPE during Cu stress. However, these two species account for less than 5% of the overall lipid content. We did not observe significant changes in any other lipid class during Cu stress or in UPEC mutant strains. Our finding indicates that FabR and FadR do not alter the ratio of phospholipid classes in the cell. However, the *ΔfabR* mutant had higher levels of polyunsaturated lipids and lower levels of saturated lipid species, whereas the *ΔfadR* mutant showed a decrease in polyunsaturated lipid content and an increase in saturated lipid content. Thus, FabR and FadR globally regulate lipid saturation states in UPEC. This observation is consistent with previous findings where FadR is required for maximal synthesis of unsaturated fatty acids, and FabR is required to regulate unsaturated fatty acid biosynthesis in commensal strains of *E. coli* (49, 51, 64, 65). These two mechanisms converge on the ability of FabR and FadR to regulate the FabAB complex (49, 51, 65). During Cu stress, WT UPEC displayed an increase in saturated lipid species. When compared to results from the TBARS assay, WT UPEC exhibited the greatest increase in MDA production. The increase in saturated lipids suggests that unsaturated lipid species are undergoing lipid peroxidation leading to cellular damage. Furthermore, the increase in polyunsaturated lipids observed in *ΔfabR* mutants may be linked to its increased sensitivity to Cu. Inversely, there are decreased levels of polyunsaturated lipids and increased levels of saturated lipids in *ΔfadR* mutant that may confer resistance to Cu stress. Cu bivalently binds to PE in the cell membrane and causes oxidation of unsaturated lipids (13). Since PE is the most abundant lipid in the *E. coli* cell, Cu could be localized to the cell membrane leading to peroxidation of lipid acyl chains and production of lipid aldehyde byproducts that can damage DNA and proteins (19, 60).

LCFA supplementation and metabolism have been shown to be strong modulators and triggers for bacterial fitness during infection (26, 27, 30, 74). Supplementation of exogenous polyunsaturated fatty acids (PUFAs), a type of LCFA, increases motility in *E. coli* (30). Therefore, *ΔfabR* and *ΔfadR* mutants were screened for irregularities in biofilm formation and swimming motility. The *ΔfabR* and *ΔfadR* mutants showed increased biofilm formation and decreased swimming motility. Initially, we hypothesized that decreased distance in swimming may be due to energy availability in fatty acid regulation mutants. During the log phase, the resazurin assay showed increased redox activity for the *ΔfadR* UPEC mutant compared to WT UPEC, but there is not a statistically significant change in redox activity relative to WT UPEC during the stationary phase. We believe that this discrepancy in redox activity could be due to constitutive degradation of LCFA in the *ΔfadR* mutant during the log phase and return to set point during the stationary phase. Therefore, we conducted a Western blot of the FliC protein which exhibited decreased production of the FliC protein in the *ΔfabR* and *ΔfadR* mutants. Our observations highlight that FabR and FadR are critical for biofilm formation and flagellin expression. Contrary to UPEC, studies in *Salmonella enterica* serovar Typhimurium show that deletion of *fadR* impairs gut colonization due to alterations in flagellar motility (74). In these studies, motility is aberrant, but the *ΔfadR* mutant exhibits larger zones of swimming than WT, and normal swimming is restored with supplementation of exogenous LCFAs (74). However, supplementation with exogenous LCFAs increases swimming motility in *E*. *coli* (30). Thus, FadR is critical for the maintenance of optimal swimming motility in many closely related bacterial species, but their phenotypic effects can vary depending on pathways involved in flagella formation.

The *in vitro* virulence assays are corroborated by the *in vivo* murine model of urinary tract infection where *ΔfabR* and *ΔfadR* mutants are attenuated in their ability to colonize and invade the host’s urinary system when co-cultured with WT UPEC. We speculate that this observation is attributed to the decreased swimming ability of *ΔfabR* and *ΔfadR* mutants. Since the *ΔfabR* and *ΔfadR* mutants have impaired swimming and previous reports have demonstrated that flagella is an important virulence factor for uropathogenesis (35, 75), the decreased fitness of the *ΔfabR* and *ΔfadR* mutants in the mouse model of UTI is likely due to the decreased expression of flagella and swimming motility. During LCFA metabolism, ubiquinone is a limiting factor for disulfide bond formation in the periplasm leading to redox imbalance and activation of the CpxRA system (20, 76, 77). Furthermore, LCFA metabolism causes reduced form of DsbA to accumulate in the periplasm hindering disulfide bond formation (77–80). Without DsbA, UPEC virulence attenuated in the murine model of acute UTI and is attributed to the disruption of *P* fimbrial and flagellar biosynthesis (79). Taken together with our findings, reduced *in vivo* fitness of the *ΔfabR* and *ΔfadR* mutants could also be triggered by a redox imbalance in the cell envelope.

A limitation of our current study is our inability to screen the effects of exogenous LCFA supplementation on survival during Cu stress. When combined with Cu, both oleic acid and sodium oleate precipitate out of the solution. We hypothesize that the combination of Cu and oleic acid derivatives forms Cu oleate, which prevents dissociation of oleic acid and Cu in solution (81). Thus, it is plausible that protection from Cu stress in *ΔfadR* mutants can be reversed by chemically complementing exogenous fatty acids to directly incorporate into the cellular envelope. The role of LCFA homeostasis on UPEC virulence should be further studied in future studies to uncover how fatty acids contribute to fimbrial biogenesis, adherence, and envelope/membrane homeostasis.

Our findings indicate a role for FabR and FadR in optimal resistance and sensitivity to Cu stress in UPEC, respectively. We also demonstrate that FabR and FadR modulate the competitive fitness of UPEC in a mouse model of UTI. In summary, our study sheds light on the previously unappreciated connection between LCFA metabolism and Cu homeostasis in UPEC.

## SUPPLEMENTAL MATERIAL

Supplemental tables and figures jb.00117-26-s0001.pdf Tables S1 to S3 and Figures S1 to S7.

Table S4 jb.00117-26-s0002.xlsx Lipidome dataset.

ASM does not own the copyrights to Supplemental Material that may be linked to, or accessed through, an article. The authors have granted ASM a non-exclusive, world-wide license to publish the Supplemental Material files. Please contact the corresponding author directly for reuse.

## References

[1] Welch RA, Burland V, Plunkett G III, Redford P, Roesch P, Rasko D, Buckles EL, Liou S-R, Boutin A, Hackett J, Stroud D, Mayhew GF, Rose DJ, Zhou S, Schwartz DC, Perna NT, Mobley HLT, Donnenberg MS, Blattner FR. 2002. Extensive mosaic structure revealed by the complete genome sequence of uropathogenic Escherichia coli*.* Proc Natl Acad Sci USA 99:17020–17024. doi:10.1073/pnas.252529799 12471157 PMC139262

[2] Flores-Mireles AL, Walker JN, Caparon M, Hultgren SJ. 2015. Urinary tract infections: epidemiology, mechanisms of infection and treatment options. Nat Rev Microbiol 13:269–284. doi:10.1038/nrmicro3432 25853778 PMC4457377

[3] Foxman B. 2014. Urinary tract infection syndromes: occurrence, recurrence, bacteriology, risk factors, and disease burden. Infect Dis Clin North Am 28:1–13. doi:10.1016/j.idc.2013.09.003 24484571

[4] Hood MI, Skaar EP. 2012. Nutritional immunity: transition metals at the pathogen-host interface. Nat Rev Microbiol 10:525–537. doi:10.1038/nrmicro2836 22796883 PMC3875331

[5] Subashchandrabose S, Mobley HLT. 2015. Back to the metal age: battle for metals at the host-pathogen interface during urinary tract infection. Metallomics 7:935–942. doi:10.1039/c4mt00329b 25677827 PMC4634365

[6] von Pein JB, Stocks CJ, Schembri MA, Kapetanovic R, Sweet MJ. 2021. An alloy of zinc and innate immunity: galvanising host defence against infection. Cell Microbiol 23:e13268. doi:10.1111/cmi.13268 32975847

[7] Hyre A, Casanova-Hampton K, Subashchandrabose S. 2021. Copper homeostatic mechanisms and their role in the virulence of Escherichia coli and Salmonella enterica. EcoSal Plus 9:eESP00142020. doi:10.1128/ecosalplus.ESP-0014-2020 34125582 PMC8669021

[8] Subashchandrabose S, Hazen TH, Brumbaugh AR, Himpsl SD, Smith SN, Ernst RD, Rasko DA, Mobley HLT. 2014. Host-specific induction of Escherichia coli fitness genes during human urinary tract infection. Proc Natl Acad Sci USA 111:18327–18332. doi:10.1073/pnas.1415959112 25489107 PMC4280598

[9] Chaturvedi KS, Hung CS, Crowley JR, Stapleton AE, Henderson JP. 2012. The siderophore yersiniabactin binds copper to protect pathogens during infection. Nat Chem Biol 8:731–736. doi:10.1038/nchembio.1020 22772152 PMC3600419

[10] Koh EI, Robinson AE, Bandara N, Rogers BE, Henderson JP. 2017. Copper import in Escherichia coli by the yersiniabactin metallophore system. Nat Chem Biol 13:1016–1021. doi:10.1038/nchembio.2441 28759019 PMC5562518

[11] Saenkham-Huntsinger P, Hyre AN, Hanson BS, Donati GL, Adams LG, Ryan C, Londoño A, Moustafa AM, Planet PJ, Subashchandrabose S. 2021. Copper resistance promotes fitness of methicillin-resistant Staphylococcus aureus during urinary tract infection. mBio 12:e0203821. doi:10.1128/mBio.02038-21 34488457 PMC8546587

[12] Saenkham P, Ritter M, Donati GL, Subashchandrabose S. 2020. Copper primes adaptation of uropathogenic Escherichia coli to superoxide stress by activating superoxide dismutases. PLoS Pathog 16:e1008856. doi:10.1371/journal.ppat.1008856 32845936 PMC7478841

[13] Poyton MF, Sendecki AM, Cong X, Cremer PS. 2016. Cu(2+) binds to phosphatidylethanolamine and increases oxidation in lipid membranes. J Am Chem Soc 138:1584–1590. doi:10.1021/jacs.5b11561 26820910

[14] Hong R, Kang TY, Michels CA, Gadura N. 2012. Membrane lipid peroxidation in copper alloy-mediated contact killing of Escherichia coli. Appl Environ Microbiol 78:1776–1784. doi:10.1128/AEM.07068-11 22247141 PMC3298164

[15] Macomber L, Imlay JA. 2009. The iron-sulfur clusters of dehydratases are primary intracellular targets of copper toxicity. Proc Natl Acad Sci USA 106:8344–8349. doi:10.1073/pnas.0812808106 19416816 PMC2688863

[16] Casanova-Hampton K, Carey A, Kassam S, Garner A, Donati GL, Thangamani S, Subashchandrabose S. 2021. A genome-wide screen reveals the involvement of enterobactin-mediated iron acquisition in Escherichia coli survival during copper stress. Metallomics 13:mfab052. doi:10.1093/mtomcs/mfab052 34415046 PMC8419524

[17] Pichot R, Watson RL, Norton IT. 2013. Phospholipids at the interface: current trends and challenges. Int J Mol Sci 14:11767–11794. doi:10.3390/ijms140611767 23736688 PMC3709755

[18] Cronan JE, Rock CO. 2008. Biosynthesis of membrane lipids. EcoSal Plus 3. doi:10.1128/ecosalplus.3.6.4 26443744

[19] Naguib M, Feldman N, Zarodkiewicz P, Shropshire H, Biamis C, El-Halfawy OM, McCain J, Dezanet C, Décout J-L, Chen Y, Cosa G, Valvano MA. 2022. An evolutionary conserved detoxification system for membrane lipid–derived peroxyl radicals in Gram-negative bacteria. PLoS Biol 20:e3001610. doi:10.1371/journal.pbio.3001610 35580139 PMC9113575

[20] Mitchell AM, Silhavy TJ. 2019. Envelope stress responses: balancing damage repair and toxicity. Nat Rev Microbiol 17:417–428. doi:10.1038/s41579-019-0199-0 31150012 PMC6596312

[21] Silhavy TJ, Kahne D, Walker S. 2010. The bacterial cell envelope. Cold Spring Harb Perspect Biol 2:a000414. doi:10.1101/cshperspect.a000414 20452953 PMC2857177

[22] Sohlenkamp C, Geiger O. 2016. Bacterial membrane lipids: diversity in structures and pathways. FEMS Microbiol Rev 40:133–159. doi:10.1093/femsre/fuv008 25862689

[23] Greenberg GR, Chakrabarti P, Khorana HG. 1976. Incorporation of fatty acids containing photosensitive groups into phospholipids of Escherichia coli*.* Proc Natl Acad Sci USA 73:86–90. doi:10.1073/pnas.73.1.86 1108021 PMC335844

[24] Parsons JB, Yao J, Frank MW, Jackson P, Rock CO. 2012. Membrane disruption by antimicrobial fatty acids releases low-molecular-weight proteins from Staphylococcus aureus. J Bacteriol 194:5294–5304. doi:10.1128/JB.00743-12 22843840 PMC3457211

[25] Cronan JE, Weisberg LJ, Allen RG. 1975. Regulation of membrane lipid synthesis in Escherichia coli. Accumulation of free fatty acids of abnormal length during inhibition of phospholipid synthesis. J Biol Chem 250:5835–5840. 1097444

[26] Mitchell MK, Ellermann M. 2022. Long chain fatty acids and virulence repression in intestinal bacterial pathogens. Front Cell Infect Microbiol 12:928503. doi:10.3389/fcimb.2022.928503 35782143 PMC9247172

[27] Ellermann M, Pacheco AR, Jimenez AG, Russell RM, Cuesta S, Kumar A, Zhu W, Vale G, Martin SA, Raj P, McDonald JG, Winter SE, Sperandio V. 2020. Endocannabinoids inhibit the induction of virulence in enteric pathogens. Cell 183:650–665. doi:10.1016/j.cell.2020.09.022 33031742 PMC7606741

[28] Jimenez AG, Ellermann M, Abbott W, Sperandio V. 2020. Diet-derived galacturonic acid regulates virulence and intestinal colonization in enterohaemorrhagic Escherichia coli and Citrobacter rodentium. Nat Microbiol 5:368–378. doi:10.1038/s41564-019-0641-0 31873206 PMC6992478

[29] Ellermann M, Jimenez AG, Pifer R, Ruiz N, Sperandio V. 2021. The canonical long-chain fatty acid sensing machinery processes arachidonic acid to inhibit virulence in enterohemorrhagic Escherichia coli*.* mBio 12:mBio doi:10.1128/mBio.03247-20 PMC7845647 33468701

[30] Herndon JL, Peters RE, Hofer RN, Simmons TB, Symes SJ, Giles DK. 2020. Exogenous polyunsaturated fatty acids (PUFAs) promote changes in growth, phospholipid composition, membrane permeability and virulence phenotypes in Escherichia coli. BMC Microbiol 20:305. doi:10.1186/s12866-020-01988-0 33046008 PMC7552566

[31] Kaper JB, Nataro JP, Mobley HL. 2004. Pathogenic Escherichia coli. Nat Rev Microbiol 2:123–140. doi:10.1038/nrmicro818 15040260

[32] Reitzer L, Zimmern P. 2019. Rapid growth and metabolism of uropathogenic Escherichia coli in relation to urine composition. Clin Microbiol Rev 33:e00101-19. doi:10.1128/CMR.00101-19 31619395 PMC6927312

[33] Baba T, Ara T, Hasegawa M, Takai Y, Okumura Y, Baba M, Datsenko KA, Tomita M, Wanner BL, Mori H. 2006. Construction of Escherichia coli K-12 in-frame, single-gene knockout mutants: the Keio collection. Mol Syst Biol 2:0008. doi:10.1038/msb4100050 PMC1681482 16738554

[34] Datsenko KA, Wanner BL. 2000. One-step inactivation of chromosomal genes in Escherichia coli K-12 using PCR products. Proc Natl Acad Sci USA 97:6640–6645. doi:10.1073/pnas.120163297 10829079 PMC18686

[35] Lane MC, Alteri CJ, Smith SN, Mobley HLT. 2007. Expression of flagella is coincident with uropathogenic Escherichia coli ascension to the upper urinary tract. Proc Natl Acad Sci USA 104:16669–16674. doi:10.1073/pnas.0607898104 17925449 PMC2034267

[36] Zaslaver A, Bren A, Ronen M, Itzkovitz S, Kikoin I, Shavit S, Liebermeister W, Surette MG, Alon U. 2006. A comprehensive library of fluorescent transcriptional reporters for Escherichia coli. Nat Methods 3:623–628. doi:10.1038/nmeth895 16862137

[37] Pfaffl MW. 2001. A new mathematical model for relative quantification in real-time RT-PCR. Nucleic Acids Res 29:e45. doi:10.1093/nar/29.9.e45 11328886 PMC55695

[38] Ohkawa H, Ohishi N, Yagi K. 1979. Assay for lipid peroxides in animal tissues by thiobarbituric acid reaction. Anal Biochem 95:351–358. doi:10.1016/0003-2697(79)90738-3 36810

[39] BLIGH EG, DYER WJ. 1959. A rapid method of total lipid extraction and purification. Can J Biochem Physiol 37:911–917. doi:10.1139/o59-099 13671378

[40] Cajka T, Fiehn O. 2014. Comprehensive analysis of lipids in biological systems by liquid chromatography-mass spectrometry. Trends Analyt Chem 61:192–206. doi:10.1016/j.trac.2014.04.017 PMC4187118 25309011

[41] Sparagna GC, Johnson CA, McCune SA, Moore RL, Murphy RC. 2005. Quantitation of cardiolipin molecular species in spontaneously hypertensive heart failure rats using electrospray ionization mass spectrometry. J Lipid Res 46:1196–1204. doi:10.1194/jlr.M500031-JLR200 15772420

[42] Rai AK, Sawasato K, Kozlova A, Sparagna GC, Bogdanov M, Mitchell AM. 2023. Differentiation of gram-negative intermembrane phospholipid transporter function by intrinsic substrate preference. bioRxiv. doi:10.1101/2023.06.21.545913 PMC11226057 38913742

[43] Fahy E, Sud M, Cotter D, Subramaniam S. 2007. LIPID MAPS online tools for LIPID research. Nucleic Acids Res 35:W606–12. doi:10.1093/nar/gkm324 17584797 PMC1933166

[44] O’Toole GA. 2011. Microtiter dish biofilm formation assay. J Vis Exp:2437. doi:10.3791/2437 21307833 PMC3182663

[45] George I, Kalairaj MS, Zimmern PE, Ware TH, Subashchandrabose S. 2024. Competitive fitness of asymptomatic bacteriuria E. coli strain 83972 against uropathogens in human urine. Infect Immun 92:e0017324. doi:10.1128/iai.00173-24 38780216 PMC11237815

[46] My L, Rekoske B, Lemke JJ, Viala JP, Gourse RL, Bouveret E. 2013. Transcription of the Escherichia coli fatty acid synthesis operon fabHDG is directly activated by FadR and inhibited by ppGpp. J Bacteriol 195:3784–3795. doi:10.1128/JB.00384-13 23772072 PMC3754556

[47] My L, Ghandour Achkar N, Viala JP, Bouveret E. 2015. Reassessment of the genetic regulation of fatty acid synthesis in Escherichia coli: global positive control by the dual functional regulator FadR. J Bacteriol 197:1862–1872. doi:10.1128/JB.00064-15 25802297 PMC4420907

[48] Campbell JW, Cronan JE Jr. 2001. Escherichia coli FadR positively regulates transcription of the fabB fatty acid biosynthetic gene. J Bacteriol 183:5982–5990. doi:10.1128/JB.183.20.5982-5990.2001 11566998 PMC99677

[49] Zhang YM, Marrakchi H, Rock CO. 2002. The FabR (YijC) transcription factor regulates unsaturated fatty acid biosynthesis in Escherichia coli. J Biol Chem 277:15558–15565. doi:10.1074/jbc.M201399200 11859088

[50] Feng Y, Cronan JE. 2011. Complex binding of the FabR repressor of bacterial unsaturated fatty acid biosynthesis to its cognate promoters. Mol Microbiol 80:195–218. doi:10.1111/j.1365-2958.2011.07564.x 21276098 PMC4072462

[51] Cronan JE. 2021. The Escherichia coli FadR transcription factor: too much of a good thing? Mol Microbiol 115:1080–1085. doi:10.1111/mmi.14663 33283913 PMC8180525

[52] Bost M, Houdart S, Oberli M, Kalonji E, Huneau JF, Margaritis I. 2016. Dietary copper and human health: current evidence and unresolved issues. J Trace Elem Med Biol 35:107–115. doi:10.1016/j.jtemb.2016.02.006 27049134

[53] Tree JJ, Ulett GC, Ong C-LY, Trott DJ, McEwan AG, Schembri MA. 2008. Trade-off between iron uptake and protection against oxidative stress: deletion of cueO promotes uropathogenic Escherichia coli virulence in a mouse model of urinary tract infection. J Bacteriol 190:6909–6912. doi:10.1128/JB.00451-08 18723628 PMC2566202

[54] Grass G, Rensing C. 2001 a. CueO is a multi-copper oxidase that confers copper tolerance in Escherichia coli. Biochem Biophys Res Commun 286:902–908. doi:10.1006/bbrc.2001.5474 11527384

[55] Grass G, Rensing C. 2001. Genes involved in copper homeostasis in Escherichia coli. J Bacteriol 183:2145–2147. doi:10.1128/JB.183.6.2145-2147.2001 11222619 PMC95116

[56] Singh SK, Grass G, Rensing C, Montfort WR. 2004. Cuprous oxidase activity of CueO from Escherichia coli. J Bacteriol 186:7815–7817. doi:10.1128/JB.186.22.7815-7817.2004 15516598 PMC524913

[57] Sokol RJ, Devereaux MW, Traber MG, Shikes RH. 1989. Copper toxicity and lipid peroxidation in isolated rat hepatocytes: effect of vitamin E. Pediatr Res 25:55–62. doi:10.1203/00006450-198901000-00014 2919118

[58] Wagner P, Heinecke JW. 1997. Copper ions promote peroxidation of low density lipoprotein lipid by binding to histidine residues of apolipoprotein B100, but they are reduced at other sites on LDL. Arterioscler Thromb Vasc Biol 17:3338–3346. doi:10.1161/01.atv.17.11.3338 9409331

[59] Kurutas EB, Ciragil P, Gul M, Kilinc M. 2005. The effects of oxidative stress in urinary tract infection. Mediators Inflamm 2005:242–244. doi:10.1155/MI.2005.242 16192676 PMC1526480

[60] Ayala A, Muñoz MF, Argüelles S. 2014. Lipid peroxidation: production, metabolism, and signaling mechanisms of malondialdehyde and 4-hydroxy-2-nonenal. Oxid Med Cell Longev 2014:360438. doi:10.1155/2014/360438 24999379 PMC4066722

[61] Terlizzi ME, Gribaudo G, Maffei ME. 2017. UroPathogenic Escherichia coli (UPEC) infections: virulence factors, bladder responses, antibiotic, and non-antibiotic antimicrobial strategies. Front Microbiol 8:1566. doi:10.3389/fmicb.2017.01566 28861072 PMC5559502

[62] Subashchandrabose S, Mobley HLT. 2015. Virulence and fitness determinants of uropathogenic Escherichia coli. Microbiol Spectr 3. doi:10.1128/microbiolspec.UTI-0015-2012 PMC4566162 26350328

[63] Koh EI, Henderson JP. 2015. Microbial copper-binding siderophores at the host-pathogen interface. J Biol Chem 290:18967–18974. doi:10.1074/jbc.R115.644328 26055720 PMC4521018

[64] Zhu K, Zhang YM, Rock CO. 2009. Transcriptional regulation of membrane lipid homeostasis in Escherichia coli. J Biol Chem 284:34880–34888. doi:10.1074/jbc.M109.068239 19854834 PMC2787350

[65] Nunn WD, Giffin K, Clark D, Cronan JE Jr. 1983. Role for fadR in unsaturated fatty acid biosynthesis in Escherichia coli. J Bacteriol 154:554–560. doi:10.1128/jb.154.2.554-560.1983 6341354 PMC217500

[66] Outten FW, Outten CE, Hale J, O’Halloran TV. 2000. Transcriptional activation of an Escherichia coli copper efflux regulon by the chromosomal MerR homologue, cueR. J Biol Chem 275:31024–31029. doi:10.1074/jbc.M006508200 10915804

[67] Joshi CS, Mora A, Felder PA, Mysorekar IU. 2021. NRF2 promotes urothelial cell response to bacterial infection by regulating reactive oxygen species and RAB27B expression. Cell Rep 37:109856. doi:10.1016/j.celrep.2021.109856 34686330

[68] Hodgkinson V, Petris MJ. 2012. Copper homeostasis at the host-pathogen interface. J Biol Chem 287:13549–13555. doi:10.1074/jbc.R111.316406 22389498 PMC3340201

[69] Saenkham-Huntsinger P, Ritter M, Donati GL, Mitchell AM, Subashchandrabose S. 2024. The inner membrane protein YhiM links copper and CpxAR envelope stress responses in uropathogenic E. coli. MBio 15:e0352223. doi:10.1128/mbio.03522-23 38470052 PMC11005409

[70] Imlay JA. 2013. The molecular mechanisms and physiological consequences of oxidative stress: lessons from a model bacterium. Nat Rev Microbiol 11:443–454. doi:10.1038/nrmicro3032 23712352 PMC4018742

[71] Nichols DS, McMeekin TA. 2002. Biomarker techniques to screen for bacteria that produce polyunsaturated fatty acids. J Microbiol Methods 48:161–170. doi:10.1016/s0167-7012(01)00320-7 11777566

[72] Zhang YM, Rock CO. 2009. Transcriptional regulation in bacterial membrane lipid synthesis. J Lipid Res 50 Suppl:S115–9. doi:10.1194/jlr.R800046-JLR200 18941141 PMC2674756

[73] Moore K, Roberts LJ. 1998. Measurement of lipid peroxidation. Free Radic Res 28:659–671. doi:10.3109/10715769809065821 9736317

[74] Hoshino Y, Sakamoto T, Sudo N, Ito M, Haneda T, Okada N, Miki T. 2022. Fatty acid homeostasis tunes flagellar motility by activating phase 2 flagellin expression, contributing to Salmonella gut colonization. Infect Immun 90:e0018422. doi:10.1128/iai.00184-22 35652649 PMC9302153

[75] Wright KJ, Seed PC, Hultgren SJ. 2005. Uropathogenic Escherichia coli flagella aid in efficient urinary tract colonization. Infect Immun 73:7657–7668. doi:10.1128/IAI.73.11.7657-7668.2005 16239570 PMC1273872

[76] Agrawal S, Jaswal K, Shiver AL, Balecha H, Patra T, Chaba R. 2017. A genome-wide screen in Escherichia coli reveals that ubiquinone is a key antioxidant for metabolism of long-chain fatty acids. J Biol Chem 292:20086–20099. doi:10.1074/jbc.M117.806240 29042439 PMC5723998

[77] Jaswal K, Shrivastava M, Roy D, Agrawal S, Chaba R. 2020. Metabolism of long-chain fatty acids affects disulfide bond formation in Escherichia coli and activates envelope stress response pathways as a combat strategy. PLoS Genet 16:e1009081. doi:10.1371/journal.pgen.1009081 33079953 PMC7598926

[78] Jaswal K, Shrivastava M, Chaba R. 2021. Revisiting long-chain fatty acid metabolism in Escherichia coli: integration with stress responses. Curr Genet 67:573–582. doi:10.1007/s00294-021-01178-z 33740112

[79] Totsika M, Heras B, Wurpel DJ, Schembri MA. 2009. Characterization of two homologous disulfide bond systems involved in virulence factor biogenesis in uropathogenic Escherichia coli CFT073. J Bacteriol 191:3901–3908. doi:10.1128/JB.00143-09 19376849 PMC2698384

[80] Heras B, Shouldice SR, Totsika M, Scanlon MJ, Schembri MA, Martin JL. 2009. DSB proteins and bacterial pathogenicity. Nat Rev Microbiol 7:215–225. doi:10.1038/nrmicro2087 19198617

[81] Low MJD, Brown KH, Inoue H. 1967. The reaction of oleic acid with copper surfaces. J Colloid Interface Sci 24:252–257. doi:10.1016/0021-9797(67)90228-7

