# Supplementary material for: Long-chain fatty acid homeostasis contributes to survival of uropathogenic E. coli during copper toxicity
Source: J Bacteriol. 2026 Mar 30;208(4):e00117-26. doi: 10.1128/jb.00117-26 (PMC13104621; doi:10.1128/jb.00117-26)
Supplement: Supplemental tables and figures — Tables S1 to S3 and Figures S1 to S7. [file jb.00117-26-s0001.pdf]

SUPPLEMENTAL MATERIAL

Supplemental Table 1. Bacterial strains used in this study.

Strains	Description[a]	Reference
E. coli		
BW25113	WT Commensal/Lab Strain	(1-3)
BW25113 *ΔfabR*	BW25113 *ΔfabR::npt*	(3)
BW25113 *ΔfadR*	BW25113 *ΔfadR::npt*	(3)
BW25113 *ΔfabR*	BW25113 *ΔfabR::npt*	(3)
BW25113 *ΔfadA*	BW25113 *ΔfadA::npt*	(3)
BW25113 *ΔfadB*	BW25113 *ΔfadB::npt*	(3)
BW25113 *ΔfadD*	BW25113 *ΔfadD::npt*	(3)
BW25113 *ΔfadE*	BW25113 *ΔfadE::npt*	(3)
BW25113 *ΔfabF*	BW25113 *ΔfabF::npt*	(3)
BW25113 *ΔfabH*	BW25113 *ΔfabH::npt*	(3)
BW25113 *ΔfadL*	BW25113 *ΔfadL::npt*	(3)
**Uropathogenic *E. coli***		
CFT073	WT UPEC	(4)
CFT073 *ΔfabR*	CFT073 *ΔfabR::cat*	This study
CFT073 *ΔfadR*	CFT073 *ΔfadR::cat*	This study
CFT073 *ΔfliC*	CFT073 *ΔfadR::npt*	(5)

[a]*npt*, neomycin phosphotransferase; *cat*, chloramphenicol acetyl transferase.

Supplemental Table 2. Plasmids used in this study.

Plasmids	Description[a]	Reference
pU66	Vector, SC101 ori, npt GFP reporter plasmid carrying gfpmut2	(6)
pU66_*cusR*	*cusR::gfp*	(6)
pU66_*cueO*	*cueO::gfp*	(6)
pU66_*fliC*	*fliC::gfp*	(6)
pGEN-MCS	Low copy number vector	(5)
pGEN_*fabR*	*fabR* complementation	This study
pGen_*fadR*	*fadR* complementation	This study

[a]*npt*, neomycin phosphotransferase.

Supplemental Table 3. Primers used in this study.

Primer Function[a]	Sequence (5’-3’)
fabR KO F	ttattgcgttaccgttcattcacaacactggagcaatccagtatggtgtaggctggagctgcttc
fabR KO R	acgctagcagcagcgtacctctatcttgatttgcttgtttcattaatgggaattagccatggtcc
fabR KO diag F	ctggatagtctgacggctcc
fabR KO diag R	acagagctgcaaaagtacca
fadR KO F	gatgagtccaactttgttttgctgtgttatggaaatctcactatggtgtaggctggagctgcttc
fadR KO R	gcatcaggcaattatacgtttgtcatccgtctggaaggattaatgggaattagccatggtcc
fadR KO diag F	gacaggagtgaggcaagtct
fadR KO diag R	acgtaggcctgataagcgta
pGEN fabR V F	ccgggaaatgtgaaggacgagtaattaccaatgcttaatcagtgagg
pGEN FabR V R	ccagcaacacgatggattgcgtggcgaaactgtcagaccaagtttactcatatatac
FabR I F	gtatatatgagtaaacttggtctgacagtttcgccacgcaatccatcgtgttgc
FabR I R	cctcactgattaagcattggtaattactcgtccttcacatttcc
pGEN fadR V F	ctgccgggtgatttagccattcagggacgataattaccaatgcttaatcagtgagg
pGEN fadR V R	ctatcaagacttgcctcactcctgtctgaccctgtcagaccaagtttactcatatatac
FadR I F	gtatatatgagtaaacttggtctgacagggtcagacaggagtgaggcaagtcttg
FadR I R	cctcactgattaagcattggtaattatcgtccctgaatggctaaatc

[a]KO, knockout; F, forward; R, reverse; V, vector; I, insert

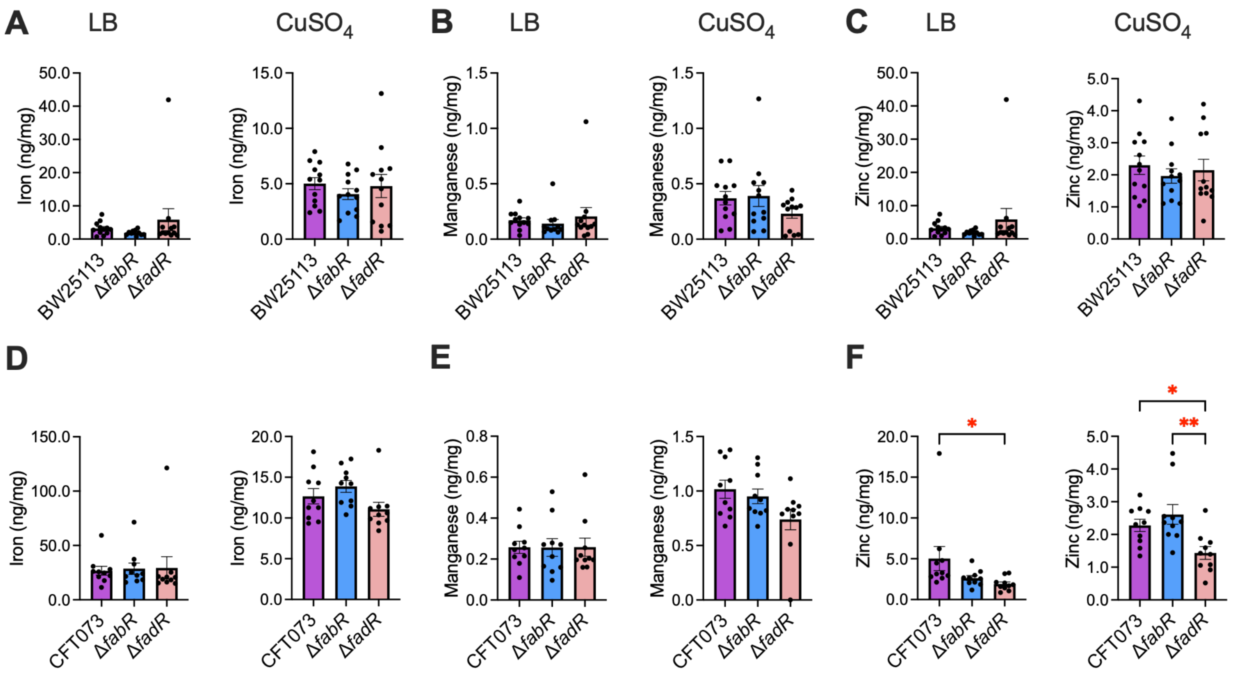

Supplemental Figure 1. Cell-associated Trace Metal Content. Commensal (**A-C**) and UPEC (**D-F**) wild-type and mutant strains were incubated in LB without (left) and with (right) Cu. Cell-associated iron (**A&D**), manganese (**B&E**), and zinc (**C&F**) were determined by ICP-MS and normalized to cell pellet weight. ANOVA with Tukey's multiple comparisons. *P<0.05 and **P<0.001

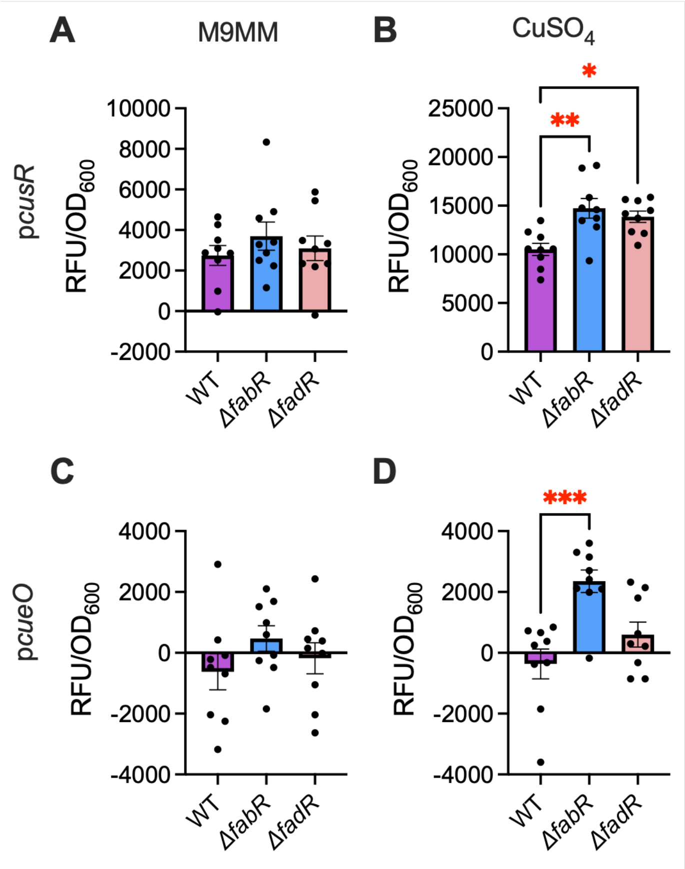

Supplemental Figure 2. Regulators of fatty acid metabolism affect *cusR* and *cueO* transcription in UPEC. WT and mutant UPEC strains transformed with CusR-regulated *cusR* and CueR-regulated *cueO* fused to the *gfp* gene were cultured in M9MM (**A** & **C**) and M9MM supplemented with 5 µM Cu (**B** & **D**). Fluorescence was determined at Ex_{485}/Em_{530} nm and normalized to optical density. Mean and SEM are plotted. ANOVA, *P<0.05, **P<0.01, and ***P<0.001.

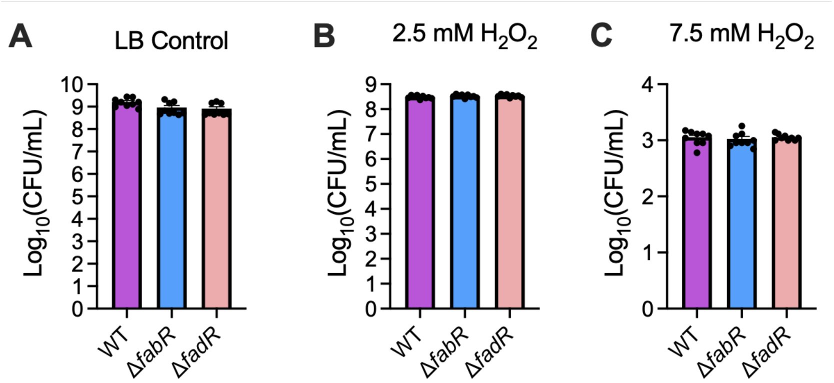

Supplemental Figure 3. Fatty acid metabolism does not contribute to hydrogen peroxide sensitivity or resistance. WT and mutant UPEC strains were cultured overnight in LB broth with 0, 2.5, and 7.5 mM H_2O_2 (A-C). CFU were enumerated and compared between strains. Assays were conducted in triplicate with at least 3 technical replicates. Results were analyzed by Kruskal-Wallis test and returned no statistical significance.

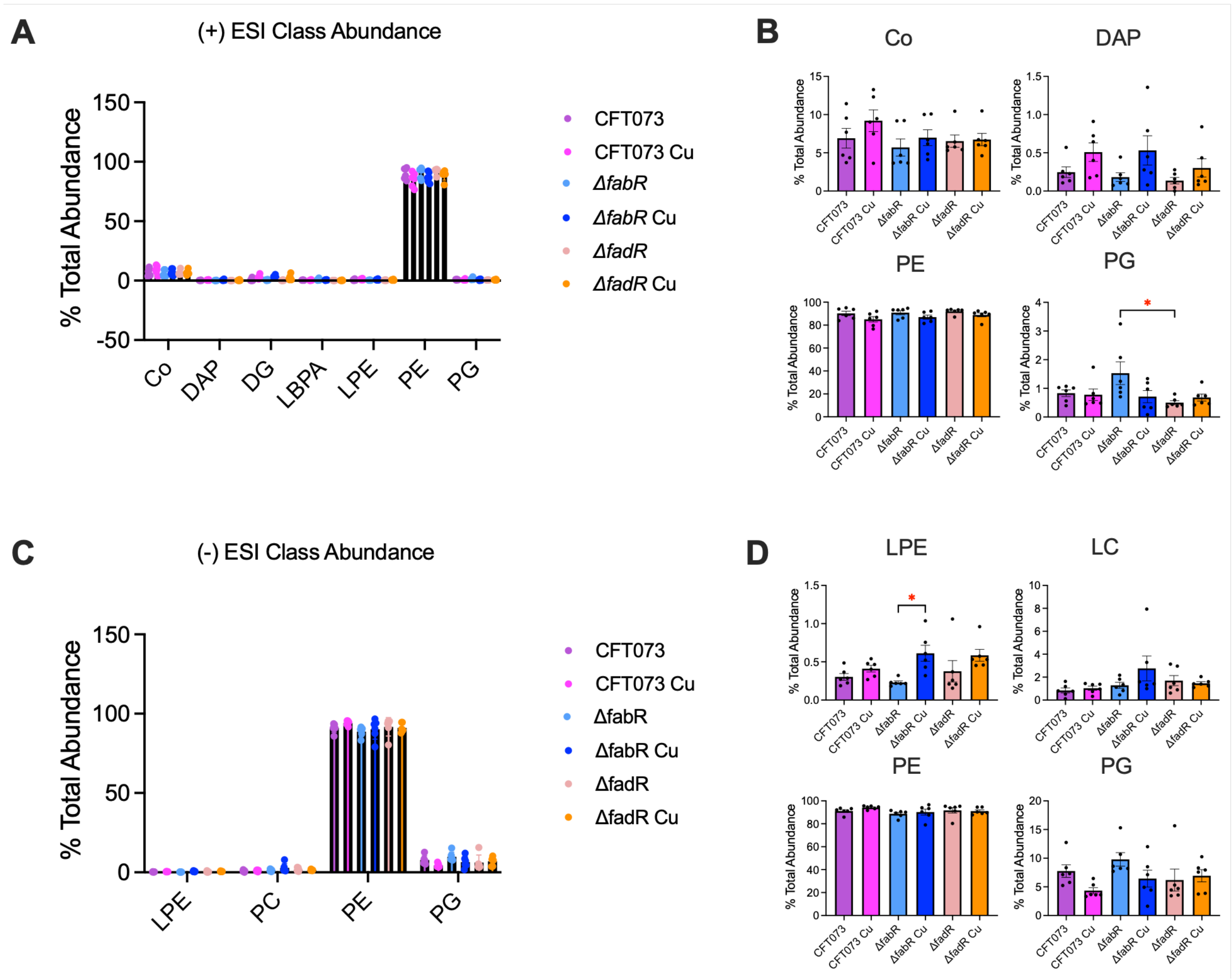

Supplemental Figure 4. Relative Abundance of Lipid Classes in UPEC. LC-MS analysis WT and mutant UPEC exposed to Cu and controls. Relative abundance of lipid classes in positive ESI mode (A&B). Relative abundance of lipid classes in negative ESI mode (C&D). Analyzed by ANOVA with tukey's multiple comparison test. *P<0.05

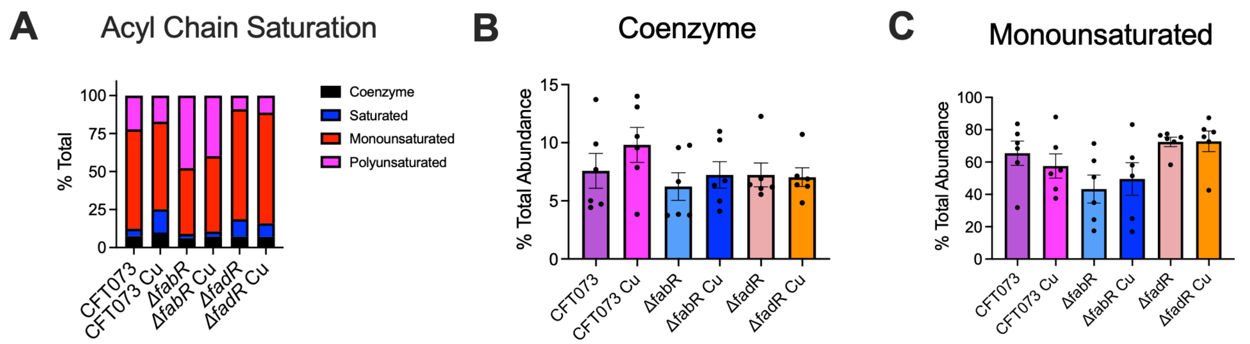

Supplemental Figure 5. Relative Abundance of Lipid Saturation levels. Relative abundance of lipid saturation states in UPEC WT and mutant strains in positive ESI mode (A-C). Results were analyzed by ANOVA and returned no statistical significance.

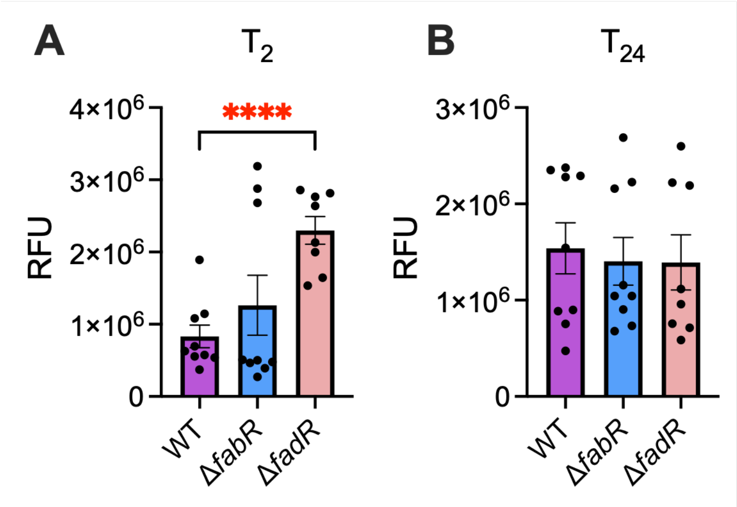

Supplemental Figure 6. FadR regulates metabolic activity during exponential growth. Starter cultures of WT and mutant UPEC strains were inoculated into black microtiter plates containing LB broth and resazurin. The plates were incubated at 37°C for 24 hours. Fluorescence was measured at 2 hours and 24 hours (excitation, 540 nm; emission, 590 nm). ANOVA with Tukey’s multiple comparisons. ****P<0.0001

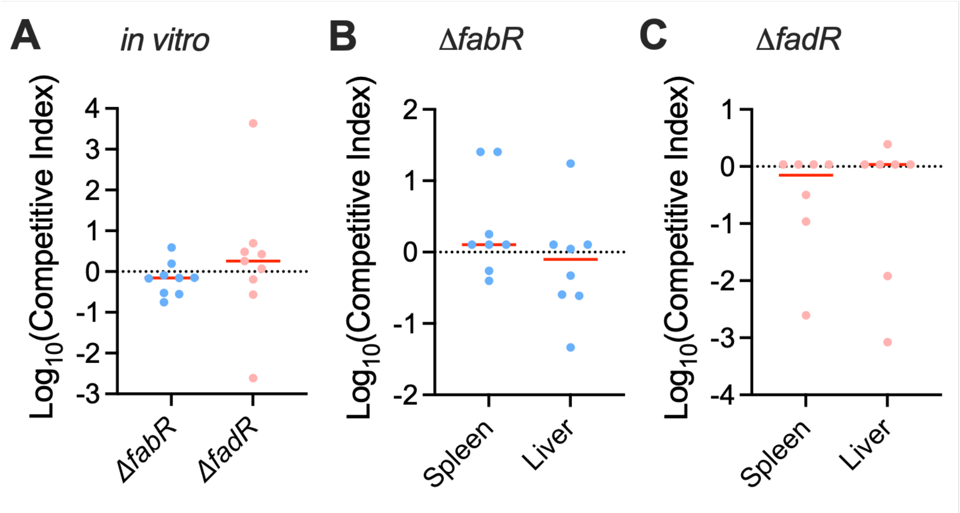

Supplemental Figure 7. In vitro Competitive index (**A)** was conducted by inoculating 1:1 ratio of WT and mutant UPEC cultures into LB broth and incubating at 37°C overnight. CFUs were enumerated and Competitive Index was calculated as the ratio of the mutant strain to the WT strain and normalized to the ratio of the inoculum. Competitive index of *ΔfabR* (**B**) and *ΔfadR* (**C**) relative to WT. Each symbol corresponds to results from one mouse and bars indicate the median. The dotted line indicates no deviation to fitness (competitive index = 1).

REFERENCES

1. Lessard IA, Pratt SD, McCafferty DG, Bussiere DE, Hutchins C, Wanner BL, Katz L, Walsh CT. 1998. Homologs of the vancomycin resistance D-Ala-D-Ala dipeptidase VanX in Streptomyces toyocaensis, Escherichia coli and Synechocystis: attributes of catalytic efficiency, stereoselectivity and regulation with implications for function. Chem Biol 5:489-504.
2. Datsenko KA, Wanner BL. 2000. One-step inactivation of chromosomal genes in Escherichia coli K-12 using PCR products. Proc Natl Acad Sci U S A 97:6640-5.
3. Baba T, Ara T, Hasegawa M, Takai Y, Okumura Y, Baba M, Datsenko KA, Tomita M, Wanner BL, Mori H. 2006. Construction of Escherichia coli K-12 in-frame, single-gene knockout mutants: the Keio collection. Mol Syst Biol 2:2006 0008.
4. Mobley HL, Green DM, Trifillis AL, Johnson DE, Chippendale GR, Lockatell CV, Jones BD, Warren JW. 1990. Pyelonephritogenic Escherichia coli and killing of cultured human renal proximal tubular epithelial cells: role of hemolysin in some strains. Infect Immun 58:1281-9.
5. Lane MC, Alteri CJ, Smith SN, Mobley HL. 2007. Expression of flagella is coincident with uropathogenic Escherichia coli ascension to the upper urinary tract. Proc Natl Acad Sci U S A 104:16669-74.
6. Zaslaver A, Bren A, Ronen M, Itzkovitz S, Kikoin I, Shavit S, Liebermeister W, Surette MG, Alon U. 2006. A comprehensive library of fluorescent transcriptional reporters for Escherichia coli. Nat Methods 3:623-8.
